# Review in coronary functional evaluation methods: applications of computational fluid dynamics and artificial intelligence in the context of coronary artery disease

**DOI:** 10.3389/fphys.2026.1875617

**Published:** 2026-10-08

**Authors:** Haojia Qu, Peiqi Lou, Yibin Pan, Debao Guan, Tianqi Wang, Hao Xu, Xiaorong Chen, Xinyang Ge

**Affiliations:** 1 College of Mathematical Medicine, Zhejiang Normal University, Jinhua, China; 2 Department of Cardiology, Jinhua Municipal Central Hospital, Jinhua, China; 3 School of Control Science and Engineering, Shandong University, Jinan, China; 4 School of Mechanical Engineering, University of Shanghai for Science and Technology, Shanghai, China; 5 Department of Medical Imaging, Jinhua Municipal Central Hospital, Jinhua, China; 6 Research and Development Department, Zhejiang Demetics Medical Technology Co., Ltd., Hangzhou, China

**Keywords:** artificial intelligence, computational fluid dynamics, coronary artery disease, functional evaluation, medical image

## Abstract

Coronary artery disease, driven by the progressive accumulation of atherosclerotic plaque within the epicardial coronary arteries, remains a leading cause of myocardial ischemia and global morbidity. A fundamental challenge in the clinical management of the disease has been the reliance on anatomic stenosis severity alone, a metric that frequently dissociates from the hemodynamic significance of a lesion and thus offers limited guidance for optimal revascularization. The advent of invasive measurements marked a transformative paradigm shift, enabling the development of a diverse functional indices for the evaluation of coronary physiology. Recently, the development of computational science and artificial intelligence has accelerated the translation of these physiological assessments into the non-invasive domain, primarily through high-fidelity numerical simulations and efficient machine learning algorithms. This review systematically examines the evolution of coronary functional assessment across several pivotal frontiers. It begins by contextualizing the diagnostic landscape prior to the widespread adoption of functional indices, where clinical decisions were predominantly informed by imaging-derived anatomical parameters. Subsequently, it traces the historical evolution of invasive coronary functional indices, critically evaluating the comparative effectiveness of pressure-derived metrics against flow-derived metrics in the detection of hemodynamically significant ischemia. Furthermore, a critical evaluation of emerging artificial intelligence methodologies for the non-invasive evaluation of functional significance were employed. By synthesizing these technological methodologies, this review aims to elucidate the appropriate clinical application of each functional index, thereby providing a road-map for clinicians to achieve precise, individualized ischemia evaluation and guide therapeutic decision-making in the contemporary era of precision cardiovascular medicine.

## 1 Introduction

Cardiovascular diseases (CVDs) represent a major category of non-communicable diseases globally, with coronary artery disease (CAD) being among the most prevalent and lethal forms, accounting for 12.8% of worldwide mortality (Hausenloy and Yellon, 2013). Accurate assessment of myocardial ischemia, serves as the cornerstone for optimal clinical decision-making and therapeutic strategy formulation (Senzaki et al., 2001). Contemporary CAD evaluation in clinical practice mainly employs two principal approaches: anatomical assessment through imaging modalities and functional evaluation of hemodynamic significance. Coronary angiography remains the gold standard for detecting epicardial coronary stenosis and guiding treatment selection between medical therapy and revascularization procedures (including percutaneous coronary intervention [PCI] and coronary artery bypass grafting [CABG]). However, the coronary circulation is located in the overall cardiovascular system of the human body and is sensitive to hemodynamic factors (Ge et al., 2021). Hemodynamic factors are involved in the entire process of CAD occurrence and development (**Figure 1**), and relying solely on imaging information is difficult to provide more critical diagnostic information. Furthermore, morphological assessment alone demonstrates significant limitations in predicting ischemia severity, particularly for moderate lesions (50-60% stenosis), necessitating complementary functional evidence to ensure appropriate revascularization outcomes. Functional assessment techniques, including coronary flow reserve (CFR) and fractional flow reserve (FFR), have consequently gained clinical prominence over several decades of development (Gould et al., 1974; Pijls et al., 1996). These methods provide critical supplemental pathophysiological information regarding myocardial ischemia, thereby enhancing objective decision-making. Recent advances in myocardial perfusion imaging, particularly positron emission tomography (PET), have further improved detection of ischemic metabolic alterations (Lo et al., 2020). The complexity of coronary pathophysiology (exemplified by the >50% prevalence of concurrent microvascular dysfunction in suspected CAD patients (Camici et al., 2015)) underscores the necessity of integrated epicardial artery and microvascular evaluation in CAD diagnosis (Kogame et al., 2020). While combined anatomical-functional assessment improves diagnostic accuracy, practical limitations including procedural complexity, substantial costs, and inconsistent results between functional indices hinder widespread clinical adoption (Johnson et al., 2012). Computational biomechanics has emerged as a transformative alternative, enabling quantitative investigation of coronary hemodynamics through sophisticated modeling approaches. This methodology facilitates both localized and system-level analyses, offering insights into disease mechanisms while circumventing the constraints of clinical measurements (Algranati et al., 2011; Taylor et al., 2013; Toba et al., 2016; Costopoulos et al., 2019). Recently, the emergence of artificial intelligence (AI) has revolutionized non-invasive FFR prediction, addressing the limitations of invasive pressure-wire measurements and computationally intensive computational fluid dynamics (CFD) simulations (Johnson et al., 2016). While invasive FFR remains the gold standard for identifying ischemia-causing lesions, its clinical adoption is hindered by procedural complexity and cost. Similarly, CFD-based FFR, despite proven diagnostic accuracy, requires extensive computational resources and prolonged processing times (Feng et al., 2024). AI methodologies, encompassing machine learning and deep learning, overcome these barriers by enabling rapid, on-site FFR evaluation directly from various imaging modalities, including coronary computed tomography angiography (CCTA) (Qiao et al., 2020), invasive angiography (Cho et al., 2019a), and intravascular imaging (Cha et al., 2020; Lee et al., 2020). By autonomously learning complex relationships between vascular morphology and hemodynamic significance from large-scale datasets, these data-driven approaches achieve diagnostic performance comparable to invasive standards while significantly reducing computational burden. Hybrid models synergizing physics-based principles with AI, such as physics-informed neural networks, further enhance fidelity and efficiency (Zhang et al., 2023). This review systematically examines the evolution, methodological innovations, and clinical implications of biomechanics research and AI-driven FFR prediction technologies.

**Figure 1:**
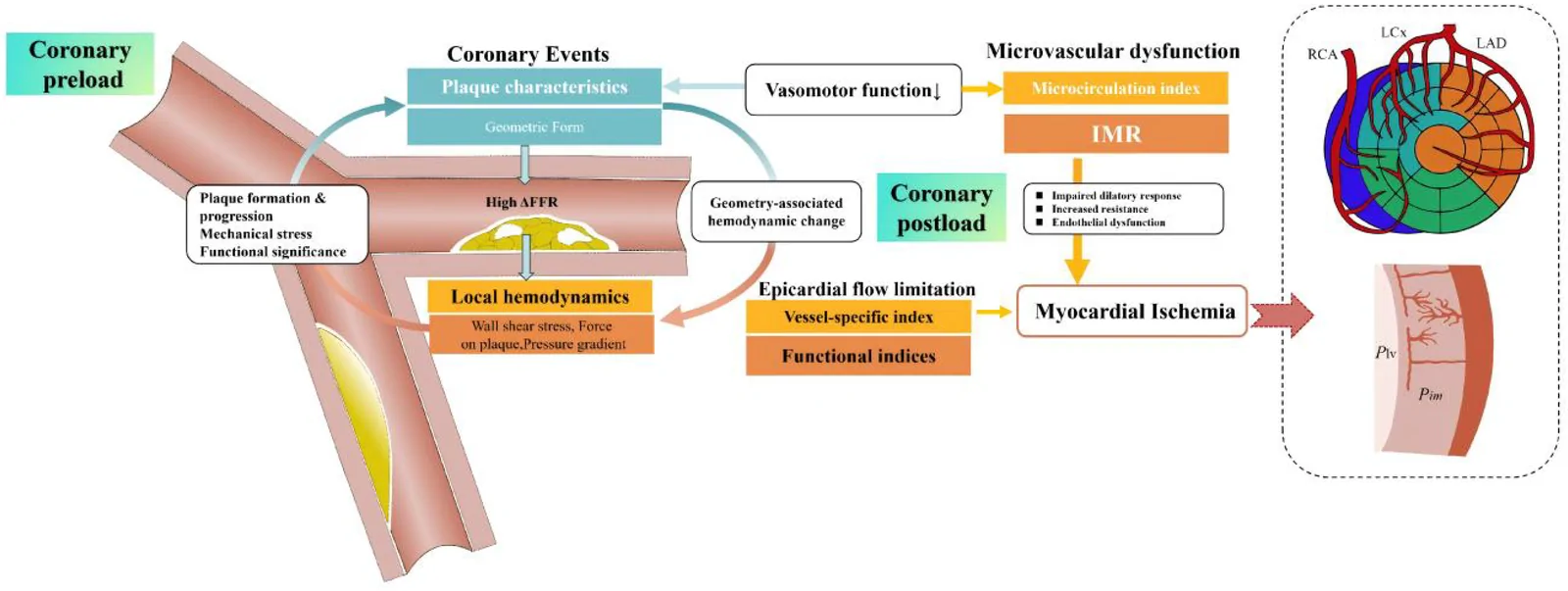
Interplay of pathophysiological hemodynamic factors and myocardial ischemia [adapted from (Yang et al., 2022)].

## 2 Search strategy and data collection

The present study conducted a systematic review of the clinical utility of coronary functional indices and the emerging potential of non-invasive assessment approaches integrating biomechanical modeling and artificial intelligence methodologies, with particular emphasis on their transformative role in precision diagnosis of coronary artery disease. A literature search of PubMed, Google Scholar, Web of Science and Baidu Scholar databases was performed from 1973 to 2026, with language of either English or Chinese, using a combination of keywords encompassing coronary artery disease, functional evaluation, coronary hemodynamics, computational fluid dynamics, medical image analysis, artificial intelligence, machine learning, deep learning, fractional flow reserve, angiography, stenosis, and related synonyms. Eligible studies included retrospective or prospective investigations reporting diagnostic accuracy of functional indices derived from both invasive and non-invasive methods. Case reports, comments, conference abstracts, and studies lacking complete diagnostic accuracy data were excluded.

## 3 Imaging diagnostic methods and current development status

### 3.1 Imaging diagnosis of epicardial coronary artery stenosis

Contemporary coronary imaging modalities include both invasive techniques (digital subtraction angiography [DSA], optical coherence tomography [OCT], intravascular ultrasound [IVUS]) and non-invasive approaches (CCTA), which facilitate visualization of coronary lumen and major branches for stenosis detection and comprehensive risk assessment. These methods evaluate multiple parameters including stenosis location, severity (percentage diameter reduction), extent (focal versus diffuse), and plaque composition, thereby informing clinical decision-making. However, despite their established role in CAD evaluation, each modality presents inherent limitations. While providing detailed morphological data, plaque characteristics, and Thrombolysis in Myocardial Infarction (TIMI) flow classification, these techniques lack quantitative capacity for assessing the amount of myocardial perfusion. CCTA offers rapid anatomical evaluation and serves as the primary screening tool for CAD, yet remains limited by wall calcifications, metallic artifacts (Iezzi et al., 2012) and unresolved challenges between radiation-dose and image-quality optimization (Zhang et al., 2021). DSA provides direct angiographic visualization, though its invasive nature and limited three-dimensional reconstruction capabilities constrain its utility (Tang et al., 2012). Intracoronary imaging modalities (IVUS/OCT) enable high-resolution cross-sectional analysis and precise plaque characterization, particularly for identifying vulnerable plaques, but their clinical adoption is hindered by procedural complexity, specialized equipment requirements, and substantial costs (Tu et al., 2011). Crucially, myocardial perfusion depends not solely on stenosis severity but rather on integrated physiological factors including hemodynamic parameters, microvascular dilation function, and intracardiac pressures (Bonow et al., 2006). This physiological complexity explains the frequent discordance between anatomical stenosis and functional ischemia, as evidenced by studies showing that significantly more stents per patient were placed in the angiography-guided group than in the FFR-guided group (2.7 ± 1.2 vs. 1.9 ± 1.3, P<0.001), demonstrating that 37% of lesions meeting anatomical criteria for intervention may not require the placement of vascular stents (Tonino et al., 2009). Conversely, functionally significant ischemia may occur with lesions below traditional anatomical thresholds (Park et al., 2012). These findings underscore the critical limitations of purely anatomical assessment and highlight the imperative for functional evaluation to complement imaging data, representing a pivotal research direction in contemporary CAD management.

### 3.2 Imaging measurement and evaluation of myocardial perfusion

The anatomical complexity of coronary circulation presents significant diagnostic challenges, with current imaging modalities capable of visualizing only approximately 5% of the coronary vascular tree, while myocardial perfusion predominantly depends on coronary microcirculation dilation function (Taqueti and Di Carli, 2018). Myocardial perfusion imaging has consequently emerged as a supplement to macrovascular assessment, providing essential clinical data for ischemic localization and quantification. Among quantitative and semi-quantitative perfusion techniques such as PET, single photon emission computed tomography imaging (SPECT), computed tomography perfusion (CTP), and magnetic resonance (MR), PET remains the reference standard (Lo et al., 2020), though its clinical utility is constrained by radiation exposure and substantial costs (Knott et al., 2019). CTP including both static and dynamic protocols, offers simultaneous assessment of coronary anatomy and myocardial perfusion, yet remains limited by radiation dose and susceptibility to motion artifacts (Seitun et al., 2018). Recent studies demonstrate that combined anatomical-functional assessment (CCTA+CTP) improves diagnostic accuracy in stented patients (4.15 ± 1.5 mSv radiation dose) compared to CCTA alone (Conte et al., 2020).

## 4 Clinical implementation of invasive coronary functional indices

Coronary functional assessment evaluates the ischemic severity induced by coronary plaques and their clinical indicative significance through hemodynamic measurements (e.g., pressure and flow), serving as a critical determinant for optimal therapeutic decision-making in stable CAD. Current clinical consensus affirms that functional index-guided revascularization optimizes the balance between medical therapy and percutaneous intervention, ultimately reducing CAD-related mortality (Neumann et al., 2019), as evidence demonstrates that approximately half of treatment decisions initially guided by anatomical assessment require reclassification (a change in the initial treatment strategy or treatment decisions by either upgrading from medical therapy to revascularization or downgrading from planned invasive angiography to conservative management) following the incorporation of FFR results into the decision-making process at the time of diagnostic angiography (Fischer et al., 2002; Van Belle et al., 2014).

CFR was first introduced in 1974 (Gould et al., 1974), represents the ratio of maximal hyperemic to resting coronary blood flow (**Figure 2**) and has been extensively validated through both non-invasive (Kern et al., 1996) and invasive techniques (Jeremias et al., 2014; Lee et al., 2017). The reported normal range of coronary flow reserve (CFR) of 3.5~5 (Barner, 1988; Baumgart et al., 1998; Windecker et al., 2002) originates from early invasive studies using epicardial pulsed-Doppler flow probes, which actually measured coronary flow velocity reserve (CFVR, the ratio of hyperemic to resting average peak velocity) rather than absolute volumetric flow reserve. Importantly, CFVR and true volume CFR are equivalent only under the assumption when the epicardial vessel cross-sectional area remains fixed. In young healthy individuals without coronary risk factors, CFVR typically exceeds 3.0-4, whereas values below this threshold are frequently observed in older populations or those with early microvascular involvement. It should be noted that the definition of a normal value for CFR is still controversial. The range of 3.5~5 applies specifically to Doppler-derived CFVR under specific experimental scenarios, and should not be generalized to measurements obtained by other techniques such as PET, CMR (quantified by myocardial perfusion reserve), or thermodilution, each of which has its own reference values and methodological caveats (Baumgart et al., 1998; Schulz et al., 2025). Methodological limitations further constrain CFR’s clinical utility: (1) Requirement for dual-state velocity measurements introduces variability from hemodynamic fluctuations (heart rate, perfusion pressure, contractility) (de Bruyne et al., 1996); (2) Absence of validated diagnostic thresholds for main vessel stenosis and the abnormal values cannot localize the pathological substrate (epicardial stenosis vs. microvascular dysfunction); and (3) Technical complexity of hyperemic induction. These factors collectively limit CFR’s widespread adoption despite its physiological comprehensiveness. FFR, currently established as the gold standard for functional assessment of coronary stenosis, evaluates hemodynamic significance through the pressure ratio across stenotic lesions during maximal hyperemia induced by vasodilators (Pijls et al., 1996) (**Figure 2**). Clinical trials have validated its diagnostic superiority, which differ substantially in follow-up duration, endpoint definitions, and the composition of composite outcomes. The FAME trial (Tonino et al., 2009) demonstrated that FFR-guided PCI significantly reduced the 1-year composite of death, myocardial infarction, or repeat revascularization compared with angiography-guided PCI (13.2% vs. 18.3%, P = 0.02), a benefit driven primarily by reductions in myocardial infarction and repeat revascularization, while all-cause mortality was similar between groups (3.0% vs. 1.8%, P = 0.19); the composite of death or myocardial infarction was also significantly lower in the FFR-guided arm (7.3% vs. 11.1%, P = 0.04). At 2-year follow-up, the benefit in the death or myocardial infarction composite was sustained (8.4% vs. 12.9%, P = 0.02), again with all-cause mortality showing no significant difference (2.6% vs. 3.8%, P = 0.25); the broader composite of death, myocardial infarction, coronary artery bypass grafting or revascularization was (17.9% vs. 22.4%, P = 0.08). At 5-year follow-up, the composite of death, myocardial infarction, or revascularization was 28% in the FFR-guided group versus 31% in the angiography-guided group (P = 0.31), with no significant difference in all-cause mortality (9% vs. 10%, P = 0.50) or the death or myocardial infarction composite (17% vs. 20%, P = 0.24). These findings indicate that the clinical benefit of FFR-guided PCI emerges early and persists primarily as a reduction in hard ischemic events and repeat revascularization, whereas the effect on all-cause mortality alone was not statistically significant at any time point. Importantly, the FFR-guided strategy used significantly fewer stents per patient than angiography-guided PCI (1.9 vs. 2.7 at 1 year), supporting the concept that selective revascularization of functionally significant lesions reduces stent-related complications without compromising long-term safety. Across all follow-up durations, the rates of death or myocardial infarction were consistently lower in the FFR-guided group, underscoring the sustained prognostic benefit of physiology-guided revascularization, while the late divergence of all-cause mortality was not observed. In the FAME 2 trial (2-year follow-up), the primary composite endpoint of death, MI, or urgent revascularization was significantly lower in the FFR-guided PCI group than in the medical-therapy group (8.1% vs. 19.5%; P<0.001) (Bruyne et al., 2014). This benefit was driven by a marked reduction in urgent revascularization (4.0% vs. 16.3%; P<0.001), whereas all-cause death (1.3% vs. 1.8%; P = 0.58) and myocardial infarction (5.8% vs. 6.8%, P = 0.56) did not differ significantly between groups (Bruyne et al., 2014). However, in a landmark analysis excluding events occurring within the first 7 days after randomization, the rate of death or MI from day 8 to 2 years was significantly lower in the PCI group (4.6% vs. 8.0%, P = 0.04). Among registry patients (FFR >0.80 in all stenoses), the 2-year primary endpoint rate was 9.0% (Bruyne et al., 2014). The DEFER trial (5-year follow-up) demonstrated that deferring revascularization in lesions with FFR ≥0.75 is safe. The composite rate of cardiac death and acute myocardial infarction was not significantly different between the deferral group and the PCI group (3.3% vs. 7.9%, P = 0.21), and event-free survival was also comparable (80% vs. 73%, P = 0.52). Notably, the risk of cardiac death or myocardial infarction related to the deferred stenosis was <1% per year and was not reduced by stenting. Furthermore, the percentage of patients free from chest pain at follow-up did not differ between the two groups (Pijls et al., 2007). In summary, the clinical benefit of FFR-guided strategies varies across endpoints and time horizons: the most consistent and pronounced effect is on urgent revascularization, the impact on the hard composite of death/MI is trial and time-dependent, and a significant reduction in all-cause mortality alone has not been demonstrated in any individual trial. However, FFR measurement remains limited by its invasive nature, procedural costs, time requirements, and potential adverse effects from hyperemic agents, resulting in clinical adoption rates below 10% (Tajeddini et al., 2020). Although the conventional threshold (FFR ≤ 0.8) reliably identifies ischemia-causing lesions (Pijls et al., 1996; De Bruyne et al., 2014), significant discordance exists with other functional metrics, demonstrating 32% diagnostic mismatch with CFR and myocardial perfusion imaging (Johnson et al., 2012; Park et al., 2012; Ahn et al., 2017).

**Figure 2:**
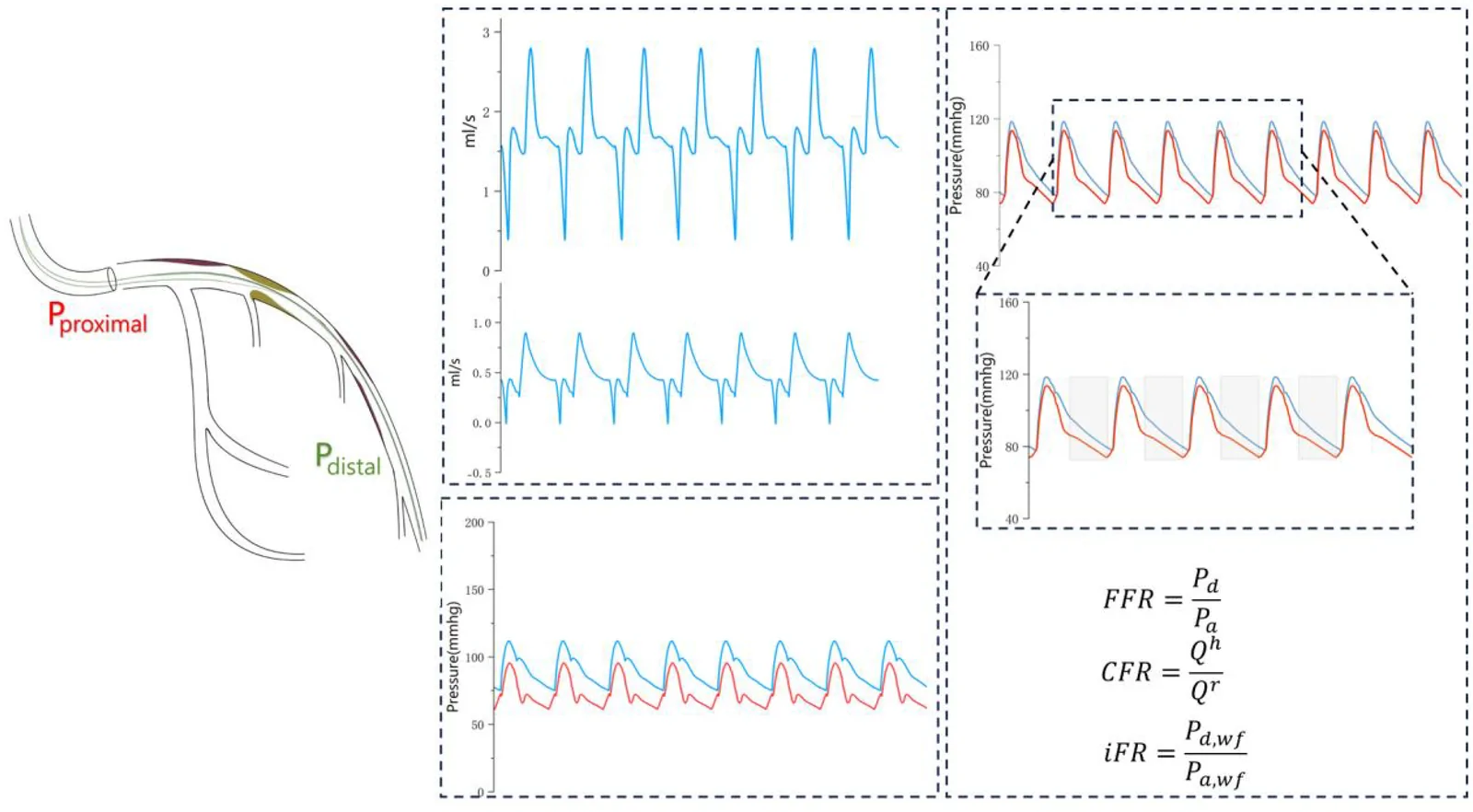
Common invasive functional evaluation indices for coronary stenosis/myocardial ischemia. *P*_d_, mean distal stenosis pressure at the maximal coronary hyperemia status; *P*_a_, mean aortic root pressure at the maximal coronary hyperemia status; CFR is the ratio of average peak velocities before (*Q*^r^) and after (*Q*^h^) adenosine; *P*_d,wf_, mean distal pressure over the wave-free period; *P*_a,wf_, mean aortic pressure over the wave-free period.

It should be noted that in the comprehensive evaluation of stenosis functional significance and myocardial ischemia, pressure-derived FFR and flow-derived CFR offer distinct and complementary diagnostic insights, with their comparative utility being profoundly influenced by underlying pathophysiological conditions. FFR exhibits superior specificity for detecting lesion-specific, focal epicardial stenosis, demonstrating a robust inverse relationship with adverse outcomes across a physiological severity continuum and proven efficacy in guiding revascularization to reduce hard endpoints such as death and myocardial infarction in randomized controlled trials. However, its diagnostic performance is notably compromised in the presence of concomitant pathologies, as elevated hyperemic microvascular resistance or significant aortic valve disease can artifactually elevate FFR, thereby masking the true hemodynamic severity of a stenosis and underestimating the extent of myocardial ischemia. Conversely, CFR, while more sensitive to a broader spectrum of factors including diffuse atherosclerosis, microvascular dysfunction, and loading conditions, offers a more comprehensive assessment of the entire coronary circulatory system, integrating the effects of both epicardial disease and microcirculatory impairment. This integrative capacity renders CFR a more reliable indicator of global myocardial ischemic burden under complex, multi-factorial pathological conditions, with stable coherence to myocardial ischemia even when FFR is misleadingly preserved, although its vulnerability to hemodynamic variations necessitates careful interpretation to avoid overestimation of epicardial stenosis severity in isolation. Thus, the optimal diagnostic strategy hinges on the clinical context: FFR remains the benchmark for guiding focal revascularization decisions, whereas CFR provides indispensable prognostic information regarding the cumulative impact of diffuse and microvascular disease, with their combined assessment offering the most comprehensive physiological characterization, particularly in patients with intermediate stenoses, suspected microvascular dysfunction, or valvular heart disease (Johnson et al., 2016; Ge et al., 2021).

The instantaneous wave-free ratio (iFR), introduced in 2012 as a vasodilator-free alternative, evaluates the diastolic pressure ratio during a wave-free period (Sen et al., 2012) (defined as the ratio of the average diastolic wave free period values of distal blood pressure and aortic blood pressure in a cardiac cycle under resting conditions, as shown in **Figure 2**). The DEFINE-FLAIR trial was designed to compare clinical outcomes between iFR and FFR-guided strategies for coronary revascularization (Davies et al., 2017; Götberg et al., 2017), demonstrating that iFR-guided coronary revascularization was non-inferior to FFR-guided revascularization with respect to the 1-year risk of major adverse cardiac events, and was associated with significantly fewer procedure-related symptoms and shorter procedural time. However, important limitations persist: iFR-FFR diagnostic consistency decreases substantially in the clinically critical range (FFR, 0.60-0.90), with relatively low agreement when using FFR as reference (Berry et al., 2013; Jeremias et al., 2014). Physiological studies reveal this discrepancy stems from differential microvascular responses, where discordant cases exhibit higher baseline microvascular resistance and greater hyperemic flow augmentation (Lee et al., 2017; Matsuo et al., 2017). Furthermore, aortic valve pathology significantly impacts iFR accuracy, though transcatheter aortic valve implantation (TAVI) can restore diagnostic performance in severe aortic stenosis (Scarsini et al., 2017; Scarsini et al., 2018). For example, for patients with severe aortic stenosis accompanied by coronary stenosis, the diagnostic accuracy rate of iFR for FFR ≤0.8 is very low (65%), but improvement was achieved after TAVI (Scarsini et al., 2018).

Beyond conventional metrics, coronary microvascular resistance (CMVR) serves as a general physiological concept that characterizes the overall resistance level of the coronary microcirculation. Multiple invasive indices have been proposed to quantitatively evaluate CMVR, among which the index of microcirculatory resistance (IMR) and hyperemic microvascular resistance (HMR) are the two most widely adopted metrics. The invasive thermodilution technique estimates flow from the mean transit time (Tmn) of a saline bolus, yielding the dimensionless index of microcirculatory resistance (IMR = P_d_×Tmn). In contrast, Doppler flow velocity measurements yield average peak velocity (APV), from which hyperemic microvascular resistance (HMR = P_d_/APV) is calculated with the unit of mmHg·cm^-^¹·s (Fawaz et al., 2023). Importantly, these two indices are not interchangeable: they are derived from different physical principles (thermodilution transit time vs. Doppler velocity), require different sensor-equipped guidewires, and have different units and diagnostic thresholds. Invasive CMVR measurements, though technically demanding and costly, reliably differentiate epicardial stenosis from microvascular dysfunction, and the measurement process requires the use of an invasive catheter that can measure both blood pressure and blood flow velocity (De Bruyne et al., 2001; Siebes et al., 2004; Aarnoudse et al., 2007). However, invasive catheters are expensive, technically difficult to operate, and the thermal dilution technique for measuring blood flow rate is prone to uncertainty errors due to a variety of factors, such as catheter positioning, injection calibration, conversion of measurement signals (Knaapen et al., 2009) and the effect of collateral circulation in the presence of main artery stenosis (Aarnoudse et al., 2004). In contrast, non-invasive CMVR is performed by combining absolute quantification of myocardial perfusion using PET, magnetic resonance imaging (CMR), or echocardiography with radiolabeled or contrast agents, measurement of mean arterial pressure via sphygmomanometry, and induction of systemic vasodilation through adenosine or dipyridamole infusion (Knaapen et al., 2009). However, the non-invasive CMVR cannot distinguish what factors (such as stenosis of the main artery, coronary microcirculation dysfunction) cause the decrease in myocardial perfusion volume.

Overall, the clinical value of the coronary microcirculation impedance indicator is mainly reflected in its complementary role to traditional coronary functional indicators such as CFR, FFR and iFR. For example, in patients with FFR above the diagnostic threshold, high CMVR indicates significant coronary microcirculation lesions, and the above information helps to comprehensively assess the main pathogenic factors of myocardial ischemia and predict the prognosis of coronary intervention (Chung et al., 2019). The inherent closed-loop architecture of the circulatory system and complex interactions between its components establish a nonlinear relationship between clinically measured hemodynamic parameters and true cardiovascular function, resulting in potential discrepancies between diagnostic test results and actual physiological status. Considering the respective limitations and advantages of current clinical assessment modalities for coronary stenosis and myocardial perfusion evaluation, biomechanical quantification emerges as a critical adjunct for elucidating the principal determinants of myocardial ischemia and their relative contributions. This approach not only establishes a theoretical framework for accurate clinical data interpretation but also facilitates personalized diagnostic assessment and therapeutic decision-making in coronary artery disease.

## 5 CFD methods for evaluating coronary functional indices

In recent decades, CFD has emerged as a pivotal tool for investigating blood flow dynamics, particularly in elucidating the relationship between hemodynamic factors and vascular disease progression. 3-Dimensional (3-D) reconstruction of coronary anatomy from clinical imaging data, coupled with CFD or fluid-solid interaction (FSI) analysis for quantitative assessment of flow characteristics and mechanical parameters (Tang et al., 2003; Eshtehardi et al., 2012; Peng et al., 2016; Liu et al., 2017; Costopoulos et al., 2019), represents a fundamental methodology for biomechanical investigation of CAD (**Figure 3**). This approach has facilitated the paradigm shift from conventional imaging-based diagnosis to integrated “imaging+mechanical” assessment frameworks (Taylor and Draney, 2004). In addition, the human coronary circulation system comprises multiple physiologically interacting components. Myocardial perfusion, a crucial parameter for assessing CAD pathophysiology, is principally governed by coronary preload (mean aortic pressure throughout the cardiac cycle), postload (intracardiac pressure exerted on coronary microcirculation), their mechanical interplay, and extracardiac factors including valvular pathology and heart rate variability (Ge et al., 2021). Systematic investigation of these determinants through computational modeling enables deeper understanding of coronary perfusion mechanisms, thereby establishing a theoretical foundation for CAD diagnosis and clinical decision-making. Coronary hemodynamic models are fundamentally constructed based on mass and momentum conservation principles (please refer to **Supplementary Appendix** for more detail). Due to the computational complexity inherent in solving 3-D governing equations within intricate coronary networks, researchers have developed dimensional simplification models including zero-dimensional (0-D), one-dimensional (1-D), and two-dimensional (2-D) models (**Figure 4**), which were tailored to specific research objectives. Current literature demonstrates distinct applications for each dimensional approach: 3-D models excel in local flow field characterization; 2-D models provide simplified analysis of axisymmetric geometries while reducing computational burden; 1-D models effectively simulate pulse wave propagation/distribution across vascular networks; and 0-D models capture macroscale pressure-flow relationships and system-level circulatory dynamics. The subsequent discussion will detail the implementation of these modeling methodologies in evaluating coronary functional indices. More details regarding model construction methods are presented in the **Supplementary Materials** document.

**Figure 3:**
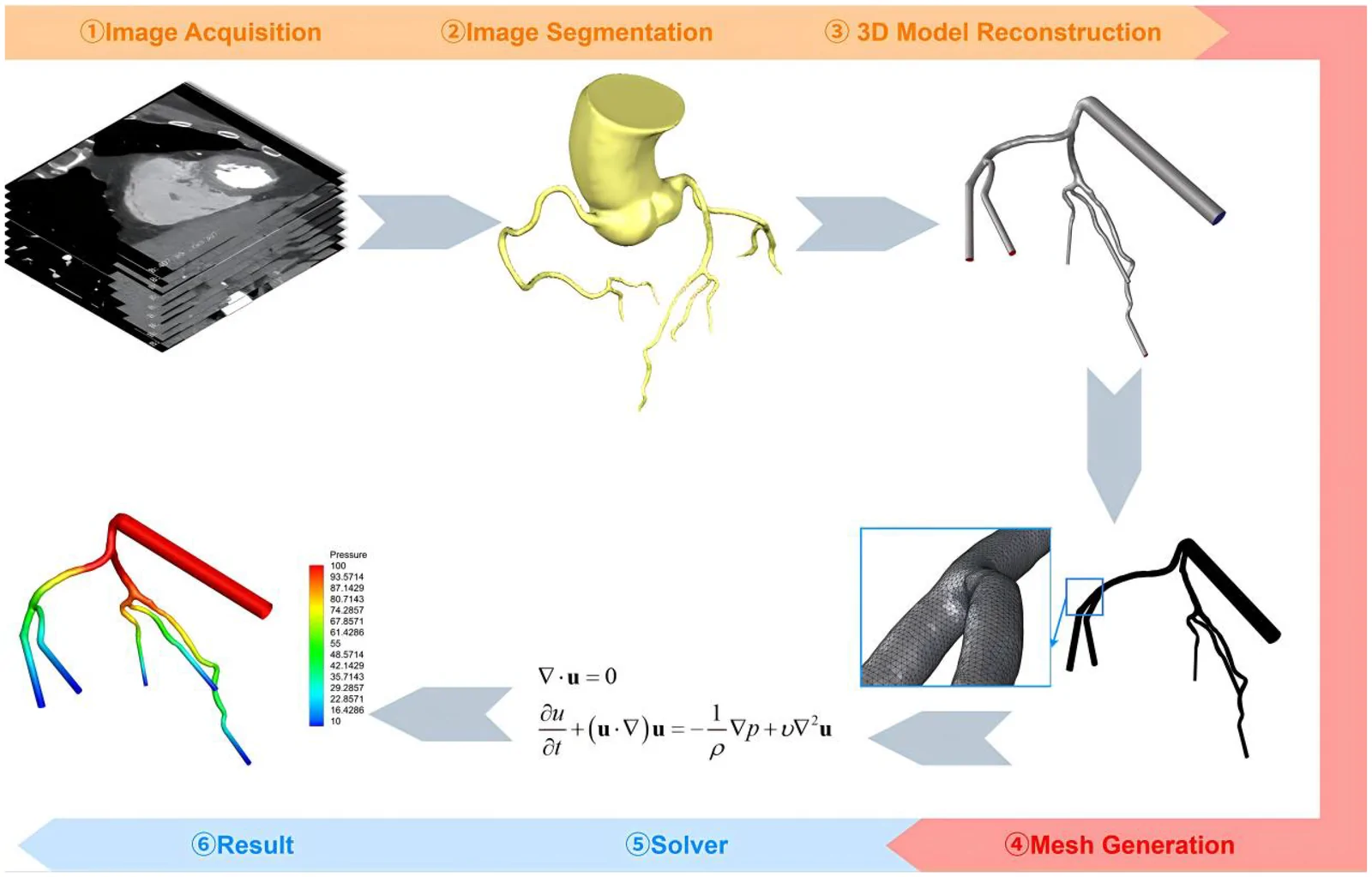
General process for constructing a 3-D coronary artery model based on medical images and CFD simulations.

**Figure 4:**
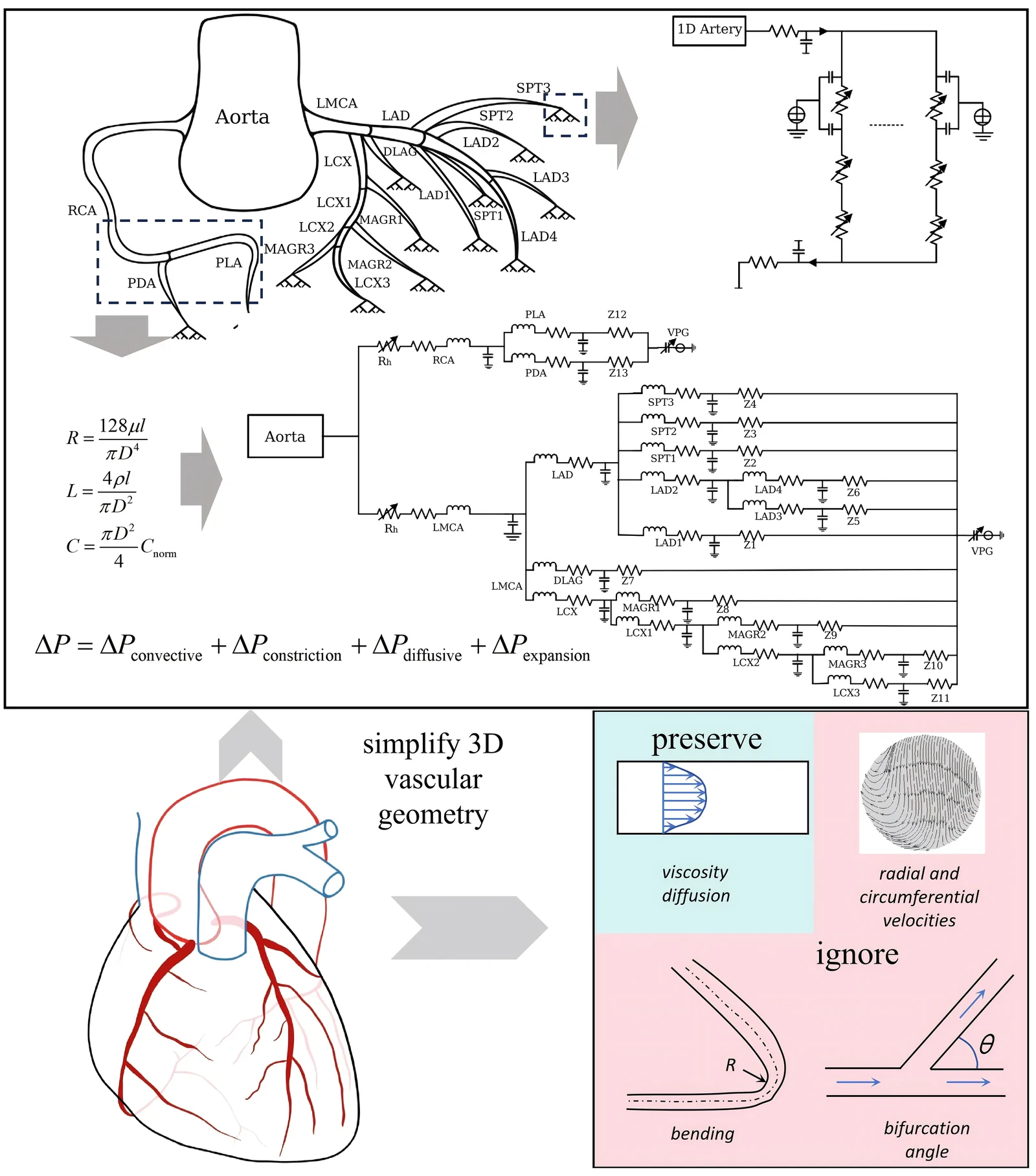
Reduced order modeling method of coronary circulation [the 0-D part was adapted from (Cai et al., 2024)].

Clinical assessment of coronary functional indices, notably FFR, has traditionally relied on invasive techniques. These methods are not only resource-intensive and time-consuming but also carry inherent risks of procedure-related complications. Consequently, over the past decades, integrating the computational models with medical imaging data to non-invasively evaluate coronary circulatory function has emerged as a prominent research focus in this field. Taylor et al. first proposed a method for estimating FFR by combining CCTA imaging data with biomechanical models (CT-FFR) (Taylor et al., 2013). Subsequently, Tu et al. advanced this approach by refining techniques in medical image analysis, geometric model reconstruction, and fluid dynamics simulation, ultimately developing the quantitative flow ratio (QFR), a more computationally efficient functional index (Tu et al., 2016). Different from traditional methods which based on CCTA for coronary artery reconstruction, this method performed from two conventional coronary angiographic projections with an angulation difference >25°using three-dimensional quantitative coronary angiography (3D QCA), which effectively corrects for foreshortening and out-of-plane eccentricity, providing the requisite vessel anatomical substrate. A frame-counting approach was used to derive contrast flow velocity for model boundary condition and a reduced-order hemodynamic model that computes the hyperemic pressure drop to derive QFR. Morris et al. developed a method for calculating virtual flow reserve fraction (vFFR) based on transient CFD analysis and rotational angiography information (Morris et al., 2013; Morris et al., 2017). The diagnostic accuracy for identifying hemodynamically significant lesions (FFR <0.80) was 97% (Morris et al., 2013). A multi-center study validated a novel FFR estimation metric based on 3-D vascular reconstruction and rapid blood flow analysis (Pellicano et al., 2017; Fearon et al., 2019). This method demonstrated high sensitivity (94%), specificity (91%), and overall diagnostic accuracy (92%) compared to invasive FFR (cutoff ≤0.80), with strong correlation (*r* = 0.80) and narrow Bland-Altman limits of agreement (-0.14 to 0.12) (Fearon et al., 2019). Recently, researchers have developed new functional evaluation indices for coronary stenosis based on the fusion of coronary imaging and CFD, such as the Virtual Functional Assessment Index (vFAI) proposed by Papafaklis et al. (2014), with diagnostic accuracy (88%), sensitivity (90%), and specificity (86%) for predicting FFR in 139 vessels from 120 patients. Overall, the primary advantage of the aforementioned methodology lies in its ability to circumvent challenges associated with the administration of vasodilators and the requirement for invasive pressure measurements. Nonetheless, the diagnostic accuracy of this approach remains subject to limitations arising from deficiencies in image information, errors in model reconstruction, and constraints imposed by computational parameters. This brings potential uncertainty to the diagnostic results. To address the limitations of CCTA in imaging coronary stenosis and plaque, researchers have proposed advanced methods that integrate high-resolution intravascular imaging techniques such as OCT and IVUS, combined with biomechanical models to assess coronary functional indices. Moreover, intravascular imaging provides detailed information on plaque composition, morphology, and endothelial characteristics, which offers significant clinical value for evaluating plaque rupture risk and informing therapeutic decision-making. Tu et al. developed and validated a novel, rapid method for computing optical coherence tomography-based fractional flow reserve (OFR) and ultrasonic flow ratio (UFR) to assess the hemodynamic significance of coronary stenoses without requiring a pressure wire or hyperemia induction (Yu et al., 2019). Both methods demonstrated excellent diagnostic accuracy (Yu et al., 2021), as shown in **Table 1**. However, both studies share several methodological constraints, including retrospective designs with modest sample sizes, which may limit statistical power and generalizability. Both studies (OFR/UFR) relied on a fixed, empirical hyperemic flow velocity that does not account for patient-specific variations in coronary flow or microvascular resistance. Additionally, potential discrepancies in measurement locations between the imaging-based computations and the invasive FFR reference were not fully reconciled, and the impact of complex vessel anatomy or prior myocardial infarction on diagnostic accuracy requires further investigation. Bezerra et al. proposed an FFR calculation method based on IVUS images and CFD analysis (Bezerra et al., 2019). The key innovations include a high-fidelity 3-D reconstruction pipeline from ECG-gated IVUS images integrated with angiography, and patient-specific boundary condition modeling based on estimated myocardial territory perfusion rather than a universal hyperemic velocity. However, its primary limitations are the small sample size, low prevalence of functionally significant stenoses, and impractical computational burden, precluding real-time clinical application. Wenxin et al. calculated iFR based on CCTA imaging data and the 0-3-D coupling method (Wang et al., 2019) to achieve non-invasive assessment of functional indicators of coronary arteries at resting state, but the main drawback was still the lack of personalized patient information in the setting of boundary conditions. Xiaolu Xi et al. first integrated both coronary vessel wall elasticity and patient-specific microcirculatory resistance (0-D model) into FFRct computation, developing and validating a physics-based 0-3-D bidirectional coupled FSI model for the noninvasive computation of fractional flow reserve from CCTA. By incorporating vascular elasticity and physiological microcirculatory boundary conditions, the model significantly enhanced the physiological fidelity and diagnostic accuracy of FFRct (Xi et al., 2022). Chulin Wu et al. first systematically quantified the impact of plaque composition on FFR within a 3-D FSI framework, highlighting the importance of considering plaque material properties in noninvasive FFR assessment. Using patient-specific coronary plaque models with FSI method, they investigated the influence of different plaque components (lipid, fibrosis, calcification) on FFR. Results demonstrated that plaque composition significantly affects computed FFR values, with differences increasing with stenosis severity (Wu et al., 2022). The FSI approach makes full use of spatiotemporal information, offering a more comprehensive perspective for evaluating the biomechanical environment of coronary lesions and underscoring its advantage over CFD models in simulating vessel motion and blood flow interactions (Fandaros et al., 2023). However, this method faces challenges such as high computational cost, numerical complexity, and convergence difficulties—large deformations of the vessel wall coupled with strong FSI can lead to mesh distortion and numerical instability, which precludes meeting clinical needs for rapid diagnosis (Bazilevs et al., 2009; Morris et al., 2015). Additionally, model parameters are sensitive and associated with considerable uncertainty; material properties of vessel walls and plaques (e.g., hyperelasticity, viscoelasticity) are difficult to measure accurately *in vivo*. These uncertainties can significantly affect the accuracy of key outcomes such as stress and strain (Torii et al., 2009), thereby limiting the widespread clinical application and adoption of FSI in routine hemodynamic assessments.

**Table 1:** Summary of CFD and FSI methods for non-invasive functional evaluation.

Methodology	Authors	Patient-derived data	image modality for geometry acquisition	Inlet/outlet boundary conditions	Main conclusion	Type of indices
3-D	Taylor et al. (2013)	N/A (Review/Methodology)	CCTA	Inlet:A lumped parameter model representing the left ventricle is coupled to the aorta at the aortic root.Outlet:a model enforces the relationship between pressure and flow.	FFRCT is superior to anatomical assessment of CTA stenosis severity in determining lesion‑specific ischemia	Pressure-based
	Bezerra et al. (2019)	24patients, 34 vessels	IVUS	Inlet: The total congestive flow calculated from patient‑specific physiological parameters serves.Outlet:A lumped‑parameter resistance model is applied at the outlet	IVUSFR is a new method that allows accurate functional evaluation of coronary stenosis, enriching the anatomical information of grayscale IVUS.	
	Morris et al. (Morris et al., 2013; Morris et al., 2017)	19 patients, 3matched datasets	ICA	Inlet: Steady flow rates of 1 ml/s and 3 ml/s to simulations Outlet: Distal impedance was tuned using measured distal pressure	vFFR showed strong correlation with measured FFR, FFR can be reliably predicted from angiographic images alone	
1-D	Pellicano et al. (2017)	184 patients, 203 lesions	ICA	Inlet: Based on the patient’s hemodynamic data.Outlet: Hyperemic flow ratio derived from an automatic resistance-based lumped model of the entire coronary tree.	high concordance between FFRangio and invasive FFR, color-coded display facilitating integration of physiology and anatomy for revascularization decision-making.	Pressure-based
	Fearon et al. (2019)	301patients, 319 vessels.	ICA	Inlet: Derived from hemodynamic data.Outlet:lumped-parameter resistance model representing the distal microcirculation.	FFRangio demonstrated high sensitivity than pressure wire-derived FFR. FFRangio may increase physiological coronary lesion assessment	
	Yu et al. (Yu et al., 2019; Yu et al., 2021)	118patients, 125 vessels	OCT	Inlet: Virtual hyperemic volumetric flow rate = proximal reference lumen area × 0.35 m/s.Outlet: Pressure drop computed via validated fluid dynamic equations modeling frictional losses and inertial losses along the stenosis.	The OCT‑based/IVUS‑based FFR calculation method is significantly superior to the traditional anatomical parameter	
		94patients, 167paired comparisons	IVUS			
	Mynard et al. (2014)	Animal studies + human anatomical data	–	Inlet: Subject-specific aortic pressure/flow and ventricular pressure formed model inputs.Outlet: Total vascular resistance determined iteratively from measured mean circumflex coronary flow	Excellent agreement was obtained between simulated and measured CxQ waveforms in most cases, No significant differences were observed in wave propagation phenomena between adults and newborns.	
	Tu et al. (2016)	73 patients,84 vessels.	ICA	Inlet: Hyperemic flow velocity modeled differently for each QFR approach.Outlet: Pressure wire-derived FFR served as the reference standard	QFR significantly improved the diagnostic accuracy of 3D-QCA-based identification of stenosis significance.	Flow-based
0-3-D	Wang et al. (Wang et al., 2019)	3D-reconstructed image of a patient	CCTA	Patient-specific lumped parameter model (LPM) boundary conditions were applied. Computations were performed at resting conditions during the wave-free period.	The computed pressure and flow of the coronary artery were more realistic. This demonstrates the feasibility of noninvasive iFR prediction from CTA images using numerical simulation.	Pressure-based
0-1-D	Sankaran et al. (Sankaran et al., 2020)	1331 patients,2,578 comparisons		Inlet: Derived from patient-specific CCTA-based modeling of coronary physiology. Outlet: Modeled using physics-driven reduced-order representation of distal microcirculation and boundary conditions.	The ROM method predicts FFRCT in real time with high accuracy compared to 3D CFD simulations.	Pressure–Flow hybrid
	Etienne Boileau et al. (2018)	no clinical patient cohort		Inlet: Pulsatile flow waveform prescribed at the inlet, scaled by a fixed cardiac output; same boundary conditions used for all cases.Outlet: Lumped-parameter coronary modelassigned to each outlet to simulate microcirculation and intramyocardial pressure.	cFFRRO showed good correlation with cFFR3D (R = 0.885, P < 0.01). Stenosis severity and shape were the most critical factors accounting for differences.	
	Carson et al. (Carson et al., 2019)	10 patients, 14 coronary lesions		4 types of inlet/outlet boundary conditions	ROMs achieved similar accuracy to 3D model. Best ROM did not require a lumped pressure-drop model. Boundary conditions and flow distribution were the main factors affecting accuracy	Pressure-based
0-D	P. Bruinsma et al. (1988)	no clinical patient cohort	–	Inlet: Coronary arterial pressure defined as a function of time; all layers share same inlet pressure; Outlet: Constant central venous pressure with time-varying intramyocardial pressure load	Models with fixed resistance and capacitance may seriously underestimate intramyocardial capacitative effects and characteristic time constants for pressure-induced resistance changes	Pressure–Flow hybrid
	Chamuleau et al. (2003)	27 patients	ICA	physiological measurement study	There is a positive association between coronary lesion severity and variability of distal microvascular resistance that normalizes after angioplasty.	
	Garcia et al. (2009)	24 patients	mathematical modeling and computational simulation study	Inlet: Coronary inlet pressure was modeled as (Pinlet−0.5·Plv− Pzf). Outlet: Microcirculatory resistance was represented through the lumped-parameter model of coronary inflow.	Valve effective orifice area was the major determinant of CFR. The impairment of CFR in AS is explained by reduced myocardial supplyand increased LV workload.	
	Dotan et al. (Algranati et al., 2010; Algranati et al., 2011)	No patient data	–	Driven by epicardial inlet and outlet pressures	Subendocardial vulnerability to acute perfusion pressure reduction stems primarily from differences in vascular compliance, Subendocardial ischemia can be improved by reduction of heart rate and left ventricular pressure	
				Inlet: Measured aortic pressure waveform. Outlet: Measured venous pressure waveformAdditional: Left ventricular pressure waveform drives CEP	Only the combined effect of chamber‑induced extracellular pressure and myocyte‑shortening‑induced intracellular pressure can achieve good agreement with the vast majority of experimental observations.	
FSI	Xiaolu Xi et al. (2022)	15 patients	CCTA	Inlet: Clinically measured patient-specific aortic pressure waveform prescribed as inlet boundary condition. Outlet: Lumped parameter model (0D) used as outlet boundary condition to incorporate physiological microcirculation	The physics-driven FSI model considers both elastic vessel wall effects and provides reliable microvascular resistance boundary conditions, enabling calculation closer to physiological conditions	Pressure–Flow hybrid
	Chulin Wu et al. (2022)	20 patients		Inlet: Lumped heart model. Outlet: Lumped coronary vascular model	Plaque composition affects vascular stiffness and vascular dilation ability, thereby affecting stenosis degree and resulting in abnormal FFR leading to myocardial ischemia	Pressure-based

In summary, while reconstructing coronary artery geometries from imaging data to simulate hemodynamic phenomena is a common practice in this field, most studies remain limited by simplified computational boundary conditions and insufficient incorporation of patient-specific physiological and pathological factors. These shortcomings represent a primary source of discrepancy between simulated results and clinically measured data. In particular, the inlet boundary conditions in many studies are often based on assumed or population-averaged flow velocity or waveform profiles, which fail to capture the true *in vivo* hemodynamic characteristics of individual patients. This limitation has been documented in studies by Molony et al. (2015) and Liu et al. (2015). Furthermore, during maximal coronary hyperemia, non-coronary factors such as aortic valve pathology and heart rate could alter coronary blood flow and consequently affect functional significance of relation between myocardial ischemia and coronary stenosis severity (Ge et al., 2021). To address this issue, a limited number of studies (Liu et al., 2015; Molony et al., 2015; Timmins et al., 2015) have employed flow boundary conditions obtained from coronary ultrasound guidewires. However, this approach is not part of routine clinical practice and therefore cannot be feasibly applied in large-scale population studies.

The key to evaluating coronary hemodynamics and functional indices based on reduced-order models lies in the rational modeling of main branch as well as coronary microcirculation. Chamuleau et al. established a single-layer intracardiac vessel model to investigate the relationship between coronary stenosis severity and myocardial microcirculatory impedance in patients with CAD (Chamuleau et al., 2003). P. Bruinsma et al. divided the myocardial coronary microcirculation network into eight layers, utilizing this model to analyze the influence of various physiological parameters on pressure-flow relationships and transmural blood flow distribution (Bruinsma et al., 1988). Garcia et al. developed a multi-layered coronary myocardial model to assess the impact of aortic valve stenosis on coronary functional indices, such as CFR (Garcia et al., 2009). Dotan et al. constructed a four-layer myocardial model to systematically examine the effects of myocardial pressure and coronary microcirculatory physiological parameters on coronary blood flow characteristics and myocardial perfusion, elucidating the mechanisms and contributing factors of inner myocardial ischemia (Algranati et al., 2010; Algranati et al., 2011). Furthermore, simplified 0-D models using algebraic formulations to evaluate stenosis pressure drop have been developed for hemodynamic simulation. The validity of stenosis modeling in reduced-order approaches is critical for accurate assessment of coronary functional indices. Given the direct correlation between FFR and coronary pressure gradients, 0-D models offer a viable approach for FFR estimation. Most recently, Sankaran et al. created a patient-specific FFR prediction model validated against 3-D simulations of over 1,300 CT datasets (Sankaran et al., 2020). The method demonstrated strong agreement with full-order CFD simulations (correlation coefficient 0.98, mean error 0.005 ± 0.015) and performed robustly across various lesion types.

Recently, Etienne Boileau et al. evaluates the accuracy of a novel reduced-order model (cFFR_RO_) based on coupled 0-1-D computational methods for the non-invasive calculation of FFR from CCTA. The core innovation lies in the application of a simplified 0-1-D model that does not require explicit stenosis or pressure-drop models, instead incorporating the geometry directly into the physical flow and pressure equations. The study concludes that this simplified, computationally efficient reduced-order model shows promise for functional stenosis assessment, potentially streamlining CAD patient management, with boundary condition selection being a critical factor for reliable pressure-drop prediction (Boileau et al., 2018). Furthermore, a benchmark study from four independent groups provides a systematic comparison of four distinct computational methodologies (three reduced-order 0-1-D models and one rigid-wall 3-D CFD model) for non-invasive evaluation of CCTA-derived FFR (cFFR) (Carson et al., 2019). Its core innovation lies in being the first publicly available, homogeneous datasets enabling direct comparison across different computational workflows, with anonymized patient data (CCTA images, clinical parameters, and invasive FFR) made openly accessible. The study reveals that, despite differing modelling assumptions (e.g., constitutive laws, boundary conditions, pressure-drop models), reduced-order models achieved diagnostic accuracy comparable to the 3-D model, with mean absolute differences ranging from 6.5% to 10.5%. The research findings emphasize that the selection of inlet/outlet boundary conditions and coronary flow distribution are the most critical factors influencing cFFR accuracy. From this perspective, reduced-order models are viable for cFFR estimation and highlights the need for standardized boundary condition strategies to improve consistency and diagnostic performance across computational platforms. Xiuhua Hu et al. (2023) introduces a novel physics-based fast model, termed FAST, for the rapid computation of blood flow and FFR in coronary arteries derived from CCTA. The key innovation lies in discretizing the coronary artery into micro-flow elements along its centerline and applying a 1-D steady-state viscous fluid motion equation that accounts for six pressure loss components: viscosity diffusion, constriction, expansion, bifurcation, bending, and flow convection. The model-results demonstrated excellent agreement with 3-D CFD and robust diagnostic performance against invasive FFR. Notably, the method achieves computation times of approximately 3 minutes per patient (dramatically faster than hours of conventional 3-D CFD methods) while maintaining high fidelity. These findings demonstrate that FAST achieves nearly real-time computational efficiency for non-invasive hemodynamic assessment and ischemia detection, showing great potential for future integration into routine diagnostic workflows and post-intervention hemodynamic simulation. Nevertheless, its current clinical applicability remains limited, given the retrospective study design, simplified equivalent diameter assumptions that may fail to represent complex irregular stenotic geometries, and the adoption of a constant bifurcation loss coefficient. Therefore, large-scale, prospective, multi-cohort validation is still essential to verify its reproducibility and generalizability before widespread clinical implementation.

In summary, numerical methods for non-invasive coronary functional assessment differ substantially in their underlying methodological complexity, ranging from simplified reduced-order models to fully coupled FSI simulations. An important methodological consideration is the substantial heterogeneity across studies included in **Table 1**. Studies differ in imaging modality, model dimensionality, boundary condition specification, reference standard, sample size, and validation strategy. In addition, performance metrics are reported inconsistently, with overall accuracy, sensitivity and specificity, and AUC or mean absolute error. These methodological differences render direct numerical comparisons between studies challenging. The values summarized in **Table 1** (main conclusion) should therefore be interpreted as illustrative of the diversity of reported performance in the current literature, rather than as directly comparable estimates of diagnostic accuracy.

## 6 Application of artificial intelligence in coronary functional assessment

Functional assessment of coronary artery stenosis represents a fundamental aspect in the diagnosis and management of CAD. Common approaches have primarily relied on invasive FFR measurements and CFD analyses. Recent advances in machine learning (ML), deep learning (DL), and physics-informed neural networks (PINN) have shown considerable promise for efficiently evaluation of coronary functional indices (**Figure 5**). This section comprehensively examines the technological advancements and clinical implementations of AI in the functional assessment of CAD and myocardial ischemia.

**Figure 5:**
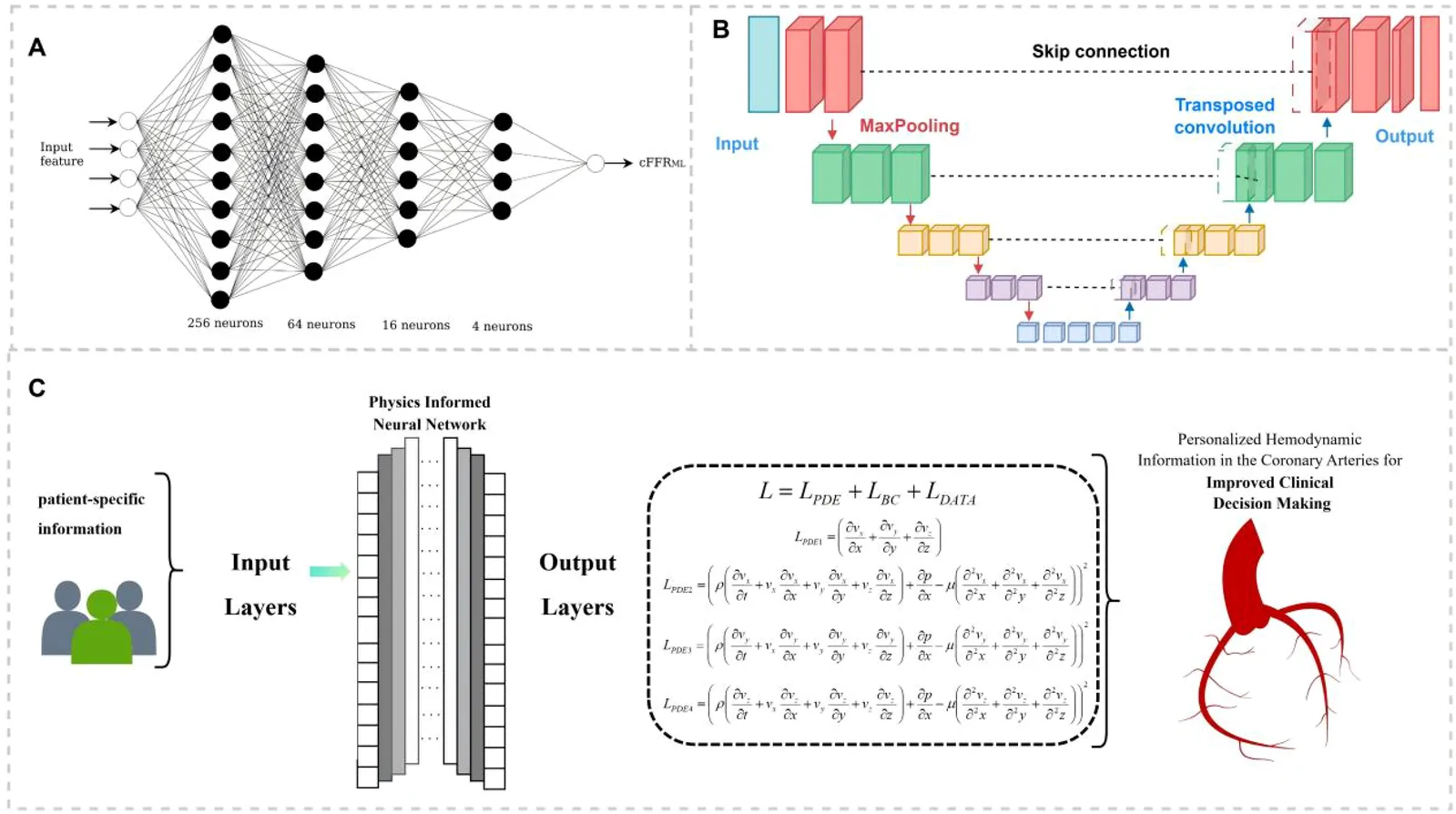
Application of AI methods in evaluating coronary functional indices. **(A)** Machine learning methods; **(B)** Deep learning methods; **(C)** Physics informed neural networks [adapted from (Itu et al., 2016; Osman et al., 2025; Sarkhosh et al., 2025)].

### 6.1 Machine-deep-learning based methods.

#### 6.1.1 Research based on CT modality

Machine learning has substantially enhanced the efficiency of functional coronary stenosis assessment by effectively integrating anatomical and hemodynamic features derived from CCTA and other imaging modalities. A prominent example is CT-derived fractional flow reserve (CT-FFR), which employs machine learning trained on CFD datasets to construct a hemodynamic surrogate model that circumvents the time-intensive CFD approach during the rapid inference stage, enabling non-invasive FFR evaluation without requiring additional diagnostic procedures. Yili Feng et al. proposed and validated a hybrid computational framework that integrates a reduced-order lumped parameter model (LPM) of the coronary and cardiovascular systems with a machine learning-based stenosis flow resistance (SFR) predictor for the rapid and accurate non-invasive computation of FFR from CCTA. The key innovation lies in coupling a closed-loop 0-D LPM, which captures the physiological interaction between the heart and circulation, with a back-propagation neural network trained on 3-D CFD-derived SFR data from idealized stenosis models. This approach avoids costly 3-D CFD simulations at the inference stage while preserving hemodynamic fidelity, it still relies on CFD-generated datasets for neural network training, rather than fully eliminating CFD from the overall modeling workflow. Accompanied by a relatively ideal prediction result (**Table 2**), the average computation time was approximately 10 minutes, representing a substantial reduction compared to conventional CFD-based FFRCT (typically hours). These findings establish the proposed FFRML method as a highly efficient and accurate tool for detecting hemodynamically significant coronary stenosis, with strong potential for real-time clinical deployment (Feng et al., 2024). Hong Yan Qiao et al. concludes that ML-based FFRCT can enhance diagnostic workflow efficiency by potentially reducing unnecessary invasive coronary angiography (ICA) referrals by 54.5% and revascularizations by 4.4%, while providing more accurate, functionally guided decision-making in stable CAD patients (Qiao et al., 2020). These findings underscore the clinical promise of ML-based FFRCT as a non-invasive tool for refining therapeutic strategies and enhancing risk stratification. However, the study is limited by its retrospective, single-center design, reliance on *post-hoc* simulations rather than prospective decision-making, lack of direct comparison with invasive FFR in most lesions, which may affect generalizability. Lee et al. employed the InceptionV3 convolutional neural network for feature extraction and integrated multiple machine learning algorithms to classify CCTA images for FFR prediction, with a focus on improving accuracy within the clinically uncertain “gray zone” (FFR 0.75-0.80). Key innovations include combining automatic coronary segmentation from CCTA with lattice Boltzmann method-based CFD simulations of both synthetic and patient-specific models to extract hemodynamic and biometric features. The multilayer perceptron achieved the highest gray zone FFR accuracy (98.9%) from internal k-fold cross-validation using a hybrid dataset of 144 patient-specific cases and 200 synthetic vessel models, with CFD-derived flow features from lattice Boltzmann simulations, representing a critical improvement over prior models. The study concludes that algorithm selection should be tailored to the clinical target (prioritizing gray zone performance over global accuracy) and that the proposed system holds promise as a reliable pre-screening tool. However, several methodological considerations should be noted when interpreting the performance metrics in the study. The model optimization was performed using outlier elimination and k-fold cross-validation, without an independent external test set. While this approach is valid for algorithm development and pre-screening purposes, the reported accuracy reflects internal validation performance rather than prospective clinical diagnostic performance in unselected populations. Independent external validation in larger, multi-center patient-specific cohorts is essential to establish generalizability before clinical implementation (Lee et al., 2022). Hyun Jung Koo et al. expanded the number of patient samples (large single-center cohort of 471 patients with 557 lesions) (Koo et al., 2021), and the primary contribution lies in its comprehensive assessment of FFRML across clinically challenging subgroups, including those stratified by coronary artery calcium score, lesion length, and minimal lumen area. However, its specificity and accuracy declined notably in lesions with smaller minimal lumen area and longer lesion length, indicating limitations in anatomically complex scenarios. Furthermore, ML-FFR exhibited reduced diagnostic accuracy for invasive FFR values near the 0.80 threshold, highlighting a vulnerability in the clinically critical “gray zone.” Meng Di Jiang et al. performed multicenter study and systematically evaluated the impact of coronary calcification on the diagnostic performance of machine learning-based CT-FFR for detecting lesion-specific ischemia, using invasive FFR as the reference standard. The high diagnostic accuracy (90%), sensitivity (84%), and specificity (94%) indicate that ML-based CT-FFR maintains robust diagnostic performance irrespective of coronary calcification burden and morphology, offering a potential solution to the longstanding limitation of CCTA in calcified lesions (Di Jiang et al., 2021), though its incomplete calcification scoring data and subjective calcification grading need to be improved and validated in a more diverse population through prospective studies. In a study conducted by Hyungjoo Cho et al., a supervised ML algorithm was developed based on XGBoost for the non-invasive identification of ischemia-producing coronary lesions, using angiographic and clinical features derived from a single projection. The model was validated on 1204 training samples using 5-fold cross-validation, demonstrating a sensitivity of 84%, specificity of 80%, overall accuracy of 82%, and an AUC of 0.87 in predicting FFR ≤ 0.80 in the test set. Though the study has limitations (such as the exclusion of complex lesions, reliance on a single angiographic view, and the lack of a continuous FFR output for granular risk stratification), the findings suggest that an angiography-based ML approach can effectively mitigate the visual-functional mismatch inherent in traditional stenosis assessment and may reduce the need for invasive pressure-wire measurements (Cho et al., 2019a).

**Table 2:** Summary of artificial intelligence-based methods for coronary artery evaluation.

Methodology	Modality	Authors (ref.)	Study design	Patients/samples	Reference standard (cutoff)	Primary endpoint/main finding
ML	CCTA	Feng Y. et al. (Feng et al., 2024)	Retrospective clinical validation (per-vessel)	91/93 lesions (27 ischemic, 29.0%)	Invasive FFR ≤ 0.80	0-D lumped-parameter model with ML-predicted stenosis flow resistance; accuracy 91.4%, AUC 0.984; ~10 min per case.
		Qiao H. Y. et al. (Qiao et al., 2020)	Retrospective, single center (consecutive enrollment, 2007–2016)	1,121	MACE follow-up (median 26 months; no per-lesion invasive FFR comparison)	FFRCT ≤ 0.80 was associated with MACE (HR 6.84); treatment regimen changed in 14.9% of patients; an FFRCT-positive strategy could reduce ICA referrals by 54.5% and revascularizations by 4.4%.
		Lee H. J. et al. (Lee et al., 2022)	Internal k-fold cross-validation	144/144 cases + 200 synthetic models	Invasive FFR (gray zone 0.75–0.80)	Accuracy of 98.9% for gray-zone FFR classification (internal validation).
		Koo H. J. et al. (Koo et al., 2021)	Retrospective, single center (large cohort)	471/557 lesions	Invasive FFR ≤ 0.80	Overall AUC 0.73; specificity and accuracy declined markedly in subgroups with small MLA and long lesions.
		Di Jiang M. et al. (Di Jiang et al., 2021)	Multicenter retrospective	442/544 vessels (344 calcified)	Invasive FFR ≤ 0.80	Accuracy 90%, sensitivity 84%, specificity 94%; impact of calcification burden analyzed.
	ICA	Cho H. et al. (Cho et al., 2019a)	Retrospective; XGBoost (12 features)	1,501/1,501 lesions (training 1,204/test 297)	Invasive FFR ≤ 0.80	Test set: sensitivity 84%, specificity 80%, accuracy 82%, AUC 0.87.
	OCT	Cha J. J. et al. (Cha et al., 2020)	Feasibility study (random forest, 36 features)	125/125 LAD lesions	Invasive FFR ≤ 0.80	Overall accuracy 95.2%.
	IVUS	Lee J.-G. et al. (Lee et al., 2020)	Retrospective (six classifiers)	1,328	Invasive FFR ≤ 0.80	Accuracy 80–83% (AUC 0.84–0.87); 85–87% after excluding gray-zone lesions.
DL	CCTA	Gao Z. et al. (Gao et al., 2020)	Trained on synthetic data, validated on clinical cases	180/13,000 synthetic + 180 real coronary trees	Invasive FFR (two criteria)	TreeVes-Net: AUC 0.92/0.93, outperforming seven baseline methods.
		van Hamersvelt R. W. et al. (van Hamersvelt et al., 2019)	Retrospective; 25–69% DS (per-patient)	101	Invasive FFR	Combined DS and DL-derived LV myocardial features: AUC 0.76 (vs. 0.68 for DS alone).
		Hampe N. et al. (Hampe et al., 2022)	Multicenter retrospective (3 hospitals)	569/514 vessels	Invasive FFR ≤ 0.80	Two-stage network (lumen regression + FFR regression); AUC 0.78.
	ICA	Arefinia F. et al. (Arefinia et al., 2024)	Retrospective;LAD angiographic frames	41/3,625 frames (50–70% stenosis)	Invasive FFR ≤ 0.80	End-to-end DenseNet169: accuracy 81%, sensitivity 86%, specificity 75%, AUC 0.81.
		Ben-Assa E. et al. (Ben-Assa et al., 2023)	Multicenter retrospective; annotation-free, view-independent (trained on >13,000 angiograms)	297/304 vessels	Invasive FFR (incl. gray zone 0.75–0.85)	Sensitivity 91%, specificity 95%, accuracy 94%; computation time 37.5 ± 7.4 s.
		Omori H. et al. (Omori et al., 2023)	Prospective (30–90% stenosis)	200/253 vessels	Invasive FFR	Accuracy 87.7%, AUC 0.90, sensitivity 76.8%, specificity 94.3%.
	OCT	Hatfaludi C.-A. et al. (Hatfaludi et al., 2022)	Retrospective; direct learning from 1-D radius sequences	80/102 lesions (LAD 57/LCX 20/RCA 25)	Invasive FFR	Accuracy 77.5% (sensitivity 72.9%, specificity 81.5%).
PINN + DL	CCTA	Zhang X. et al. (Zhang et al., 2023)	Trained and validated on simulation data	220 aortic CFD models	CFD simulation (FFRCT, WSS)	PointNet/PointNet++ with PINN embedding of the Navier–Stokes equations; rapid 4-D hemodynamic prediction.
PINN	Simulation	Alzhanov N. et al. (Alzhanov et al., 2024)	Simulation validation (small sample)	3 coronary trees	CFD and invasive FFR (error 1.2–2.8%)	3-D PINN simulates coronary flow without outlet boundary conditions and predicts FFR.
	DSA	Xie B. et al. (Xie et al., 2025)	Retrospective	184/215 vessels	Invasive FFR	BVPINO derives continuous Angio-FFR curves from angiography, capturing the trans-stenotic pressure drop.
	Simulation	Wong H. S. et al. (Wong et al., 2024)	Synthetic data; multi-case architecture comparison	parameterized 2-D stenotic tube models	CFD benchmark	Comparison of Mixed/Hypernetwork/Modes architectures; the Modes architecture performed best.
	CCTA/ICA	Chen X. et al. (Chen et al., 2025)	Boundary-progressive training (label-free)	22/22 coronary geometries	Invasive FFR	Accuracy 90.9% (vs. 81.8% for the vanilla PINN); <3 min per case.
	4D Flow MRI	Kissas G. et al. (Kissas et al., 2020)	1-D PINN; partial supervision	1 volunteer + benchmark cases	*In vivo* 4D Flow MRI	Noninvasive prediction of the arterial pressure field; Windkessel parameters calibrated in post-processing.
	Simulation	Arzani A. et al. (Arzani et al., 2021)	No boundary conditions; sparse interior data	idealized 2-D/3-D stenosis and aneurysm models	CFD benchmark	Near-wall flow and WSS reconstructed from sparse velocity data.
	MRA + TCD	Sarabian M. et al. (Sarabian et al., 2022)	Partial supervision; fusion of sparse clinical measurements	23 Circle-of-Willis segments	3D TOF-MRA + TCD (validated against 4D Flow MRI)	Reconstruction of cerebral hemodynamics; inverse updating of vessel diameters enables vasospasm detection.
	Simulation	Chen X. et al. (Chen et al., 2026)	Differentiable coordinate transformation	numerical benchmarks	CFD benchmark	Unit-domain mapping with Jacobian-based hard constraints; markedly improved simulation accuracy in stenotic vessels.
	—	Wang S. et al. (Wang et al., 2023)	Training-strategy guideline	—	—	PINN training strategies: loss-term weighting, causal sampling, and NTK-based spectral-bias analysis.

End-to-end deep learning models, including DenseNet and U-Net, automate the extraction of features from CCTA images, thereby enabling direct FFR estimation without the need for manual annotation. For instance, a DenseNet169-based DL-FFR model was proposed by Farhad Arefinia et al., achieving an accuracy of 81%, sensitivity of 86%, specificity of 75%, and an AUC of 0.81 in classifying lesions as FFR > 0.80 or ≤ 0.80. The model’s end-to-end design eliminates the need for manual feature annotation or integration of clinical variables, offering a streamlined, automated approach suitable for real-time use in catheterization laboratories without workflow disruption (Arefinia et al., 2024). Zhifan Gao et al. introduces TreeVes-Net, a novel deep learning framework designed to infer FFR directly from static CCTA images, thereby enabling non-invasive assessment of hemodynamically significant coronary stenosis. The model employs a tree-structured recurrent neural network with bidirectional long short-term memory (LSTM) units to capture spatial dependencies of blood flow dynamics along the coronary tree, effectively encoding the physical constraints imposed by the Navier-Stokes equations. To overcome the scarcity of labeled clinical data, the authors generated 13,000 synthetic coronary trees with corresponding FFR labels via 1-D hemodynamic simulations, which were used to train the network. Validation on 180 real clinical cases demonstrated strong agreement with invasive pressure-wire measurements (**Table 2**). The framework outperformed seven other machine learning approaches, including random forest, support vector machine (SVM), and single-path LSTM, underscoring the value of incorporating anatomical structure and bidirectional information flow. Nevertheless, the reliance on synthetic training data, while necessary, may not fully capture the heterogeneity of real-world coronary pathology. Additionally, the model’s performance depends on accurate coronary segmentation, and the relatively modest size of the clinical validation cohort limits generalizability (Gao et al., 2020). In a separate study, van Hamersvelt et al. demonstrated a hybrid diagnostic framework that integrates conventional coronary degree of stenosis (DS) assessment with DL-based analysis of the left ventricular myocardium (LVM) from resting CCTA to improve the identification of patients with functionally significant coronary artery stenosis. Patients with intermediate stenosis (25–69% DS, n = 101) were subjected to fully automatic DL-based characterization of the LVM using a convolutional auto-encoder, followed by classification via a SVM. The combined approach yielded an AUC of 0.76, significantly improving upon DS evaluation alone (AUC = 0.68). Specificity increased from 31.1% (using a ≥50% DS threshold) to 48.4%, albeit with a modest reduction in sensitivity from 92.6% to 84.6%. These findings suggest that DL-based myocardial analysis can provide complementary functional information in the diagnostically challenging subset of patients with intermediate coronary stenosis, potentially reducing unnecessary invasive referrals (van Hamersvelt et al., 2019). However, multicenter studies with larger cohorts and prospective validation are warranted to confirm its clinical utility and generalizability. Nils Hampe et al. presents a deep learning method for non-invasively predicting functionally significant coronary artery stenosis (FFR ≤ 0.8) from CCTA without requiring manual artery segmentation at test time. The method first extracts a multi-planar reformation of each coronary artery and uses a convolutional neural network to regress per-cross-section lumen area, mean lumen attenuation, and calcium area. These characteristics are combined with bifurcation and main-branch/side-branch indicators derived from the coronary tree and fed into a second network that performs both FFR regression and binary classification of hemodynamic significance (Hampe et al., 2022).The results outperform previous deep learning approaches that avoid coronary artery segmentation, suggesting the method could reduce unnecessary invasive FFR measurements.

#### 6.1.2 Research based on ICA modality

CCTA-based approaches for AI-derived FFR enable non-invasive physiological assessment from a single diagnostic scan, whereas ICA-based approaches derive FFR from routine invasive angiography without the need for a pressure wire or hyperemic agent. These two modalities differ fundamentally in image acquisition (non-invasive vs. invasive), temporal resolution, and clinical workflow. In parallel with CCTA-based approaches, several AI-derived FFR methods have been developed using ICA as the input imaging modality. Unlike CCTA-based FFR, which is obtained prior to invasive catheterization, ICA-FFR is computed during the diagnostic angiogram and can therefore directly inform same-session revascularization decisions.

Recent years, angiography-derived FFR techniques have emerged as an option, with AI approaches offering the potential for automated, real-time computation without altering catheterization laboratory workflow. The AutocathFFR system, a deep learning-based platform trained on over 13,000 angiographic procedures, demonstrated sensitivity of 91%, specificity of 95%, and overall accuracy of 94% in a multicenter retrospective study of 304 vessels, with a computation time of 37.5 ± 7.4 seconds, and maintained high accuracy in the FFR gray zone (0.75–0.85) (Roguin et al., 2021; Ben-Assa et al., 2023). Similarly, the angioFFR system reported accuracy of 87.7% and area under the curve (AUC) of 0.90 in 253 vessels using invasive FFR as the reference standard (Omori et al., 2023). Beyond deep learning, an XGBoost model using 12 angiographic features derived from single-projection diameter plots achieved sensitivity of 84%, specificity of 80%, and AUC of 0.87 in 297 lesions (Cho et al., 2019b), while a transfer learning approach with DenseNet169 for left anterior descending artery lesions reported accuracy of 81% and AUC of 0.81 (Arefinia et al., 2024). The ongoing MPFFR pivotal trial is prospectively validating a novel AI-based angiography-derived FFR algorithm (Chu et al., 2025), while the QUITE RIGHT study demonstrated that Murray law-based QFR (μQFR)-guided virtual percutaneous coronary intervention was superior to angiography-guided intervention in achieving optimal post-procedural physiological outcomes (absolute difference 9.1%, P<0.001) (Chen et al., 2024). In conclusion, ICA based AI-FFR represents a promising paradigm for physiological coronary assessment, combining the anatomical detail of invasive angiography with functional insight without the logistical burden of pressure wire-based measurement. However, prospective outcome trials are essential to establish their clinical role. The reviewed studies share several methodological limitations. (1) Most studies are retrospective, single-center, or modestly sized, with limited external validation. (2) A considerable portion of studies excluded complex anatomies, which restricted the generalizability. (3) Most platforms were developed on specific vessel territories (predominantly LAD) and require prospective, multicenter outcome trials to confirm clinical utility and generalizability before routine adoption.

#### 6.1.3 Research based on multi medical imaging modalities

Beyond computed tomography, higher-resolution intravascular imaging modalities such as OCT combined with real-time angiographic data provide multidimensional input for ML models, enabling simultaneous functional ischemia assessment. Jung-Joon Cha et al. presented a novel machine learning framework that integrates intravascular OCT-derived morphological features with clinical variables to predict FFR in patients with intermediate coronary stenosis of the left anterior descending artery. A total of 125 patients were enrolled, with a Random Forest algorithm trained on 36 features, including minimal lumen area, percent area stenosis, lesion length, and clinical parameters such as platelet count and hypertension, achieved strong predictive performance (overall accuracy of 95.2%). The model outperformed prior CFD-based OCT-FFR approaches and offered the advantage of simultaneous anatomical and functional assessment from a single invasive procedure, potentially reducing procedural time and cost. However, the generalizability to other coronary territories remains to be validated (Cha et al., 2020). A deep learning-based approach for predicting FFR values through analysis of raw, reduced-order anatomical data extracted from OCT images was proposed by Hatfaludi et al. A key contribution of this work is the demonstration that deep neural networks can learn predictive features directly from raw 1-D radius sequences without the need for hand-crafted feature engineering. Furthermore, the study revealed a quasi-linear relationship between dataset size and model accuracy, suggesting that performance could be further improved with larger training cohorts (Hatfaludi et al., 2022).

June-Goo Lee et al. performed a comprehensive evaluation of IVUS-based ML models for predicting FFR in intermediate coronary artery lesions in a large cohort of 1,328 patients with non-left main lesions. Using an automatic segmentation model, 99 computed IVUS features were extracted and combined with six clinical variables to train six binary classifiers. In the test set, all ML models demonstrated robust diagnostic performance for identifying ischemia-producing lesions (FFR ≤ 0.80), with overall accuracies ranging from 80% to 83% and AUCs between 0.84 and 0.87. Notably, after excluding lesions in the FFR gray zone (0.75-0.80), accuracy improved to 85-87%. The study demonstrates that ML can effectively integrate complex morphological and clinical data to overcome the limitations of traditional IVUS criteria, potentially reducing the need for invasive pressure wires (Lee et al., 2020).

### 6.2 Application of PINN in coronary functional assessment and cardiovascular hemodynamics

Physics-informed neural networks (PINNs) have emerged as a powerful paradigm for integrating physical conservation laws (by embedding the Navier-Stokes equations directly into the neural network’s loss function (Chan et al., 2025)) with sparse clinical measurements in cardiovascular flow modeling. Unlike conventional CFD, which requires complete specification of boundary conditions and expensive mesh generation, PINNs embed the governing partial differential equations as soft constraints in the loss function, enabling solutions from incomplete data. This approach allows the network to learn not only from labeled CFD-derived data but also from the underlying physical laws governing blood flow. By penalizing deviations from the governing partial differential equations, PINNs ensure that the predicted velocity and pressure fields are physically consistent, even in regions with sparse data or complex flow patterns. This hybrid data-physics paradigm overcomes key limitations of purely data-driven models, such as poor generalization to unseen geometries or turbulent regimes, and enables real-time, accurate 4-D hemodynamic mapping from vascular morphology and time inputs alone. Cardiovascular PINN applications have employed both full Navier-Stokes equations and reduced-order models. The study performed by Xuelan Zhang et al. presented a novel deep learning framework integrating PINNs with point cloud-based architectures to enable rapid, accurate, and non-invasive prediction of 4-D hemodynamics (3-D velocity and pressure fields evolving over a cardiac cycle) in patient-specific vascular models. The key innovation lies in coupling a multi-attribute point cloud dataset-derived from CFD simulations of 220 patient-specific aortic models (with and without fine coronary branches), with customized PointNet and PointNet++ architectures, and further enhanced by PINNs that embed the Navier-Stokes equations into the loss function to enforce physical consistency. The framework accurately reproduces clinically relevant metrics of FFRCT and wall shear stress. These findings establish PINN-augmented deep learning as a powerful tool for real-time hemodynamic assessment, with strong potential for clinical integration in surgical planning and ischemia diagnosis (Zhang et al., 2023). Nursultan Alzhanov et al. proposed a 3-D PINN framework that embeds the Navier-Stokes equations into its loss function to simulate coronary blood flow and predict FFR without requiring explicit outlet boundary conditions. When trained with CFD data, the model achieved excellent agreement with invasive FFR (errors: 1.2-2.8%) and demonstrated superior computational efficiency over traditional CFD. However, the purely physics-driven model exhibited poor generalizability (errors up to 29%), and the study’s small sample size and reliance on simulated data underscore the need for validation on larger, real-world clinical cohorts before translation into practice (Alzhanov et al., 2024). Baihong Xie et al. (2025) introduces a bi-variational physics-informed neural operator for predicting continuous FFR curves from coronary angiography. The key innovation lies in incorporating a variational mechanism that constrains both basis function learning and residual evaluation using local weighting functions, thereby enabling the network to capture fine-grained stenosis features such as across-stenosis pressure descent and post-stenosis recovery. Wong et al. (2024) solved steady incompressible Navier–Stokes in parameterized 2D stenotic tube models, using tube-specific coordinates (distance-to-wall and centerline distance) as auxiliary inputs to enhance the network’s ability to resolve near-wall gradients. Their work systematically compared three multi-case architectures, namely Mixed Network, Hypernetwork, and Modes Network, demonstrating that the Modes architecture, analogous to DeepONet, achieved superior accuracy, convergence efficiency, and computational speed when pre-trained on a range of geometric parameters and generalized to unseen stenosis morphologies. Chen et al. (2025) extended this paradigm to patient-specific coronary angiography data, introducing a boundary complexity metric that quantifies geometric irregularity through stenosis severity, wall curvature, and length ratio. Their boundary-progressive PINN (BP-PINN) incrementally transitions from simplified to complex geometries during training, leveraging pretrained model weights to achieve per-case computation times under three minutes and non-invasive FFR prediction with 90.9% diagnostic accuracy against invasive measurements. Arzani et al. (2021) addressed a fundamentally different challenge: reconstructing near-wall hemodynamics and wall shear stress from extremely sparse velocity measurements located away from the vessel wall. Critically, their formulation operates without knowledge of inlet or outlet boundary conditions, demonstrating that the physical constraints encoded in the Navier–Stokes equations, combined with sparse interior data, suffice to recover accurate near-wall flow features in both 2D and 3D idealized stenosis and aneurysm models. Complementing these approaches, reduced-order PINN formulations have demonstrated remarkable capability for network-level hemodynamic prediction with substantially lower computational costs. Kissas et al. (2020) and Sarabian et al. (2022) employed 1D pulse wave propagation equations (coupling mass and momentum conservation with a pressure-area elastic constitutive law) to model blood flow in arterial networks represented as graph topologies. This reduced-order formulation enables the simulation of entire vascular trees, such as the Circle of Willis comprising 23 vessel segments, while preserving physiologically relevant wave dynamics. A pivotal advantage of these 1D formulations is their ability to operate without prescribed inlet or outlet boundary conditions; interface loss functions enforcing mass and momentum conservation at bifurcations allow information to propagate across the network from sparse internal measurements alone. Kissas et al. (2020) further demonstrated that once trained, PINN predictions can be post-processed to calibrate Windkessel model parameters at each outlet (a task that is prohibitively costly in conventional CFD workflow) while providing a computationally efficient pathway to subject-specific boundary condition identification.

The training supervision strategies across these studies span a spectrum from fully unsupervised to clinically supervised. Wong et al. (2024) and Chen et al. (2025) trained the model entirely without labelled data, relying exclusively on the residuals of the governing equations and boundary conditions as loss functions. In contrast, Kissas et al. (2020) and Sarabian et al. (2022) integrated partially supervised learning, combining physical constraints with clinical measurements (by velocity and cross-sectional area from 4D Flow MRI or transcranial Doppler ultrasound) to infer pressure waveforms that cannot be measured non-invasively. Sarabian et al. further introduced an Area Surrogate PINN (ASPINN) to estimate vessel cross-sectional area time series from velocity data alone, overcoming the clinical unavailability of wall displacement measurements in cerebral arteries. This framework successfully reconstructed cerebral hemodynamics from only routinely available data (3D time-of-flight MR angiography and transcranial doppler velocity at nine locations) and demonstrated clinical utility in detecting cerebral vasospasm by solving an inverse problem to update local vessel diameters. Validation strategies likewise span from synthetic CFD benchmarks (Arzani et al., 2021; Wong et al., 2024) to *in vivo* 4D Flow MRI (Kissas et al., 2020) and invasive FFR measurements (Chen et al., 2025), with the latter achieving the highest level of clinical corroboration.

Despite these advances, several limitations warrant consideration. First, validation has predominantly been against CFD or limited clinical data rather than prospective clinical outcomes, and generalizability across heterogeneous patient populations remains to be established. Second, cross-case generalization strategies (whether multi-case pre-training, boundary-progressive transfer learning, or network-level graph formulations) differ fundamentally in their assumptions and applicability, and no unified framework yet exists. Third, the substantial training costs which ranging from several hours to over 36 hours for complex networks remain a barrier to routine clinical adoption, though transfer learning and reduced-order formulations offer promising pathways to mitigate this limitation. Finally, the fundamental challenge confronting PINN-based cardiovascular modeling lies in structuring the optimization landscape to accommodate geometric irregularity, competing physical constraints, and temporal causality. The spectral bias, which preferentially learns low-frequency features while failing to capture sharp gradients in stenotic jets and near-wall shear layers (Wang et al., 2023). This is compounded by pronounced imbalance among competing loss terms (PDE residuals, boundary conditions, and initial conditions) that differ markedly in scale and gradient magnitude, biasing optimization toward certain constraints at the expense of physical consistency. Additionally, transient simulations are undermined by causality violation, as standard PINNs minimize residuals at all time levels simultaneously, contravening the forward propagation of physical information (Wang et al., 2023). Patient-specific vascular geometries introduce further obstacles: inconsistent normalization in anisotropic coordinates, inaccurate boundary enforcement, and geometric amplification of loss imbalance, a difficulty that recent advances in differentiable coordinate transformation and causality-aware training have begun to systematically address. For instance, the JacobiNet framework proposed by Xi Chen et al. addresses these through differentiable coordinate transformation to a regular reference domain, with automatic Jacobian computation enabling hard boundary constraints, reducing relative L_2_errors in stenosed vessel simulations (Chen et al., 2026). Collectively, these methodological advances (spectral bias mitigation, causality-respecting training, and geometry-aware coordinate transformation) offer a coherent pathway toward robust PINN-based cardiovascular modeling, though computational cost and prospective validation remain significant challenges.

In summary, machine learning and deep learning have revolutionized coronary functional assessment by enabling rapid, non-invasive, and accurate prediction of FFR and ischemia detection across multiple imaging modalities, with hybrid physics-data approaches achieving diagnostic performance comparable to invasive standards while reducing computation time from hours to seconds. Current applications span from handcrafted feature-based models to end-to-end architectures, successfully addressing clinically challenging scenarios such as the FFR gray zone and demonstrating superior prognostic value over anatomical assessment alone. However, It should be noted that the performance metrics summarized in **Table 2** should be interpreted with caution and not as directly comparable across studies. This field is characterized by substantial methodological heterogeneity including studies differ in AI algorithm architecture, reference standard (invasive FFR with specific cutoffs), unit of analysis (per-patient vs. per-vessel), validation strategy (internal vs. external vs. multi-center), training datasets characteristics, and endpoint definitions. Therefore, direct accuracy comparisons between studies are methodologically inappropriate. The values in **Table 2** are presented to illustrate the range and diversity of reported performance in the literature, not to establish a hierarchy of diagnostic accuracy. It is encouraged to evaluate each study’s performance within its specific methodological context, with particular attention to validation strategy and reference standard. The future trajectory points toward seamless clinical integration through fully automated pipelines, multi-modal data fusion incorporating anatomical, hemodynamic, and plaque characteristics, and continued advancement of physics-informed neural networks that synergize data-driven learning with fundamental physiological principles, ultimately positioning these techniques as indispensable tools for precision cardiovascular medicine pending large-scale prospective validation.

## 7 Conclusion

In conclusion, the landscape of coronary functional assessment has been fundamentally reshaped by the advent and convergence of four distinct yet interrelated computational paradigms, each contributing unique capabilities to the armamentarium against coronary artery disease. CFD has matured from a purely research-oriented tool into a clinically validated methodology, enabling the derivation of FFR from standard angiographic and CCTA datasets with escalating fidelity, thereby circumventing the need for invasive pressure wires and pharmacologic hyperemia. Building upon this foundation, AI has emerged as a transformative force, with machine and deep learning models demonstrating remarkable prowess in automating feature extraction, deciphering complex anatomical-hemodynamic relationships, and achieving diagnostic performance that, in many instances, rivals or even surpasses conventional CFD approaches, all while operating with unparalleled computational efficiency. Concurrently, FSI modeling has advanced the frontier by moving beyond rigid vessel assumptions to interrogate the coupled biomechanics between blood flow and the compliant arterial wall, offering deeper insights into the mechanobiological drivers of plaque progression and vulnerability, albeit with commensurate increases in computational complexity. Most recently, the development of integrated multiscale models, which synergistically couple zero-/one-dimensional circulatory models with three-dimensional representations and incorporate patient-specific microvascular resistance, represents a pivotal stride toward holistic, personalized physiology, capturing the intricate interplay between epicardial conductance and myocardial perfusion. Collectively, these technological trajectories are not merely parallel advancements but are increasingly converging into a cohesive paradigm of precision cardiovascular medicine. The path forward lies in the synergistic fusion of physics-based fidelity with data-driven efficiency, rigorous prospective multi-center validation to establish clinical efficacy and safety, and the seamless integration of these powerful computational tools into intuitive, real-time clinical workflows, thereby empowering clinicians to deliver truly individualized, physiology-guided care.
