# Supplementary material for: Review in coronary functional evaluation methods: applications of computational fluid dynamics and artificial intelligence in the context of coronary artery disease
Source: Front Physiol. 2026 Oct 8;17:1875617. doi: 10.3389/fphys.2026.1875617 (PMC13687798; doi:10.3389/fphys.2026.1875617)
Supplement: Supplementary file 1 [file SupplementaryFile1.docx]

Appendix

（A1）Application of 3-D Model in Evaluating Functional Indicators

3-D model-based coronary artery reconstruction and functional assessment hold significant clinical value. The general workflow of this approach comprises the following key steps:

(a) Medical Image Acquisition and 3D Reconstruction:

Patient-specific coronary artery geometries are extracted from CCTA or invasive coronary angiography data using image segmentation techniques, followed by reconstruction of a three-dimensional coronary tree model that includes major branches^[12]^.

(b) Mesh Generation and Boundary Condition Specification:

The 3D geometric model is discretized into a computational mesh, and physiologically relevant boundary conditions are applied. In prior studies, the inlet boundary is often prescribed using population-averaged or personalized flow velocity/rate profiles. At the outlets, a LPM, described in detail later) is typically adopted to represent coronary microvascular resistance. By adjusting distal microcirculatory resistance, maximal hyperemia (commonly induced by pharmacological agents such as adenosine) can be simulated, which is a prerequisite for computing FFR^[127]^.

(c) Hemodynamic Simulation:

Under the prescribed boundary conditions, blood flow and pressure distributions within the coronary arteries are simulated by solving the Navier–Stokes equations^[128]^.

(d) CT-FFR Computation and Validation:

The simulated ratio of distal coronary pressure to aortic pressure yields the noninvasive FFR estimate, termed CT-FFR. Its diagnostic performance is evaluated by comparison with invasively measured FFR using a pressure wire (the clinical gold standard, with a commonly used ischemic threshold of <0.80) in the same patient. Key validation metrics include sensitivity, specificity, and the area under the receiver operating characteristic curve AUC^[129]^.

Notably, this methodology requires only routine resting CCTA data, without the need for additional scanning or pharmacological stress. By integrating 3-D anatomical reconstruction with CFD simulation, it provides functional insights noninvasively. Multiple studies have demonstrated that, compared with anatomical stenosis assessment alone, CT-FFR significantly improves diagnostic specificity and more accurately identifies ischemia-causing lesions, thereby supporting optimized clinical decision-making^[128, 129]^.

The governing equation of the 3D hemodynamic model is the classical Navier Stokes equation:

|  | $\begin{matrix} \nabla\cdot\mathbf{u}=0 \\ \frac{\partial u}{\partial t}+\left(\mathbf{u}\cdot\nabla\right)\mathbf{u}=-\frac{1}{\rho}\nabla p+\upsilon\nabla^{2}\mathbf{u} \end{matrix}$ | (1) |
| --- | --- | --- |

Herein, t is time, **u** is velocity vector, *ρ* is blood density, *p* is pressure, and *υ* is kinematic viscosity of blood.

（A2）Application of fluid structure interaction method in coronary artery model

Fluid-structure interaction methods have been widely applied in coronary hemodynamic simulations. The governing equations for the three-dimensional hemodynamic model are the classical Navier–Stokes equations. In the fluid domain, blood flow is considered as an incompressible, viscous Newtonian fluid under laminar flow conditions, governed by the Navier–Stokes equations:

$$\text{∇⋅}\text{u}\text{=0}\text{}$$

$$\text{ρ}\left( \frac{\text{∂u}}{\text{∂}\text{t}}\text{+}\left( \text{u⋅∇} \right)\text{u} \right)\text{=∇⋅}\left[ \text{-}\text{p}\text{I+κ} \right]\text{+F}$$

(2)

where $\text{t}$ denotes time, u is the velocity vector, $\text{p}$ is the pressure, $\text{ρ}$ is the blood density, $\text{κ=}\text{μ}\left( \text{∇u+∇}\text{u}^{\text{s}} \right)$, *μ* is the dynamic viscosity, and $\text{F}$ is the body force.

In the solid domain, the vessel wall structure satisfies the mechanical equilibrium equation:

$$\text{ρ}_{\text{s}}\frac{\text{∂}\text{u}_{\text{s}}}{\text{∂}\text{t}}\text{=∇⋅}\text{σ}_{\text{s}}\text{+F}$$

(3)

where $\text{ρ}_{\text{s}}$ is the vessel wall density, $\text{u}_{\text{s}}$ is the vessel wall velocity, $\text{σ}_{\text{s}}$ is the stress tensor and $\text{F}$ is the body force.

Different from rigid wall models, FSI simulations require handling dynamically deforming vessel boundaries, which is generally realized through the Arbitrary Lagrangian-Eulerian (ALE) framework. Differ from pure Lagrangian or Eulerian descriptions, the ALE framework effectively allows the computational mesh to move arbitrarily (independent of the material motion), enabling flexible mesh adaptation and accurate boundary tracking during vascular wall deformation. At the interface, mesh nodes follow the displacement of the solid wall; within the fluid domain, internal mesh nodes are smoothly updated using mesh‑smoothing algorithms to maintain element quality while preserving the topology. At the fluid-solid interface, two fundamental coupling conditions must be strictly satisfied to ensure physical consistency. Kinematic coupling conditions enforce the continuity of displacement and velocity between the fluid and solid boundaries. Dynamic coupling conditions guarantee stress equilibrium across the interface, ensuring mechanical force balance between blood flow and vessel wall tissue. The ALE framework provides the kinematic description of grid motion that enables the enforcement of the physical coupling conditions at the interface.

（A3）2-D model

3-D computational fluid dynamics (3-D CFD) offers significant advantages in capturing detailed flow field characteristics, albeit at the expense of substantial computational resources. To optimize computational efficiency while maintaining applicability to specific hemodynamic problems, previous studies have developed various reduced-order models (2-D, 1-D, and 0-D) through targeted simplifications of 3-D models. Two-dimensional models are primarily employed for local hemodynamic analysis in axially symmetric geometries. Gashi et al. demonstrated that 2-D models could predict FFR with errors within clinically acceptable ranges^[130]^. Ghigo et al. developed an innovative 2-D method that eliminates the conventional requirement for assumed cross-sectional velocity profiles, thereby enabling accurate capture of flow separation phenomena. Furthermore, the model could capture the flow behaviors in the presence of pathologies such as stenoses, in particular flow recirculation, downstream of the stenosis^[131]^, which may provide a usefull and computational resource saving platform as a reliable alternative to 3-D models.

（A4）1-D mathematical model

One-dimensional and zero-dimensional models exhibit particular advantages for rapid simulation of complex hemodynamic phenomena in coronary trees and myocardial tissue. The 1-D hemodynamic model sacrifices 3-D vascular morphology while preserving lumen diameter variations along the vascular axis. Through numerical solution of the 1-D hemodynamic equations and vessel pressure-volume relationships, this approach simulates intravascular blood flow and pressure wave propagation/reflection. Compared to 3-D models, 1-D models require substantially fewer computational resources while maintaining rapid processing speeds, with previous studies confirming their capability to accurately describe pressure wave propagation and blood flow distribution in arterial systems^[132-134]^.

The one-dimensional governing equations for coronary arterial blood flow are derived from the classical mass and momentum conservation principles^[133]^. These equations are obtained by integrating the three-dimensional Navier-Stokes equations over the vascular cross-section^[135]^, based on specific assumptions regarding coronary hemodynamics (blood pressure is uniformly distributed across the cross-section and that the axial velocity component dominates, rendering the radial velocity component negligible).

|  | $\frac{\partial A}{\partial t}+\frac{\partial Q}{\partial z}=0$ | (4) |
| --- | --- | --- |
|  | $\frac{\partial Q}{\partial t}+\frac{\partial}{\partial z}\left(\gamma\frac{Q^{2}}{A}\right)+\frac{A}{\rho}\frac{\partial P}{\partial z}+F_{r}\frac{Q}{A}=0$ | (5) |

Herein, t is time, x represents the xxial coordinates of blood vessels, *ρ* is blood density (1.06 g/cm^3^), A is the cross-section area, Q is the volumetric flow rate, P the blood pressure, *γ* is the momentum correction coefficient, and *F*_r_ is the friction force per unit length of blood vessels. Blood viscosity is divided into two categories: Newtonian fluid model and non Newtonian fluid model^[86, 136, 137]^.

In order to derive a closed form of equations 3 and 4, it is necessary to introduce a pressure volume relationship equation^[132]^ that describes the viscoelastic deformation of the blood vessel wall.

|  |    | (6) |
| --- | --- | --- |

Here, *P*_0_ is the reference blood pressure, *E*_h_ is the Young's modulus, *h* is the thickness of vessel wall, *σ* is the poisson's ratio, *r*_0_ is the radius of the vessel when *P* = *P*_0_, *τ*_σ_, and *τ*_ε_ represent the stress and strain relaxation time constants characterizing vascular viscoelasticity, respectively^[138]^. Mass flow conservation and blood pressure continuity equations at all arterial bifurcations were applied to correlate the hemodynamic variables of adjacent arteries for blood flow at the branching points of blood vessels^[133]^.

（A5）0-D mathematical model

The 0-D model represents a further simplification of the 1-D approach, eliminating spatial variations of physical quantities along the vascular axis and converting the governing equations from partial differential to ordinary differential form. This transformation reduces numerical complexity and computational demands. Structurally analogous to electrical circuits, 0-D models (exemplified by the Windkessel model) establish direct correlations between hemodynamic parameters (pressure, flow rate, vascular impedance, blood inertia, and vessel elasticity) and electrical components (voltage, current, resistance, inductance, and capacitance)^[139, 140]^. This formulation enables characterization of complex vascular network hemodynamics using basic circuit principles with resistor-capacitor networks. Such an approach not only dramatically reduces model complexity but also minimizes reliance on difficult-to-measure microcirculatory geometric parameters during model construction. Young and Tsai^[141]^ established pioneering reduced-order pressure drop models through steady-state and unsteady-state in vitro experiments, providing empirical parameter values. Seeley and Young^[142]^ subsequently refined the model to better incorporate geometric determinants of pressure drop, validating its performance through experimental studies. However, the semi-empirical nature of these models and their reliance on multiple empirical parameters may limit reliability across diverse stenosis geometries and hemodynamic conditions. To overcome these limitations, Huo et al. developed an empirical parameter-free pressure drop model derived from the Bernoulli equation, accounting for various energy losses along the vessel^[143]^, though its accuracy remains dependent on stenosis length parameters. Alternative approaches include various reduced-order formulations and regression-based models that predict pressure drops from stenosis geometry and flow velocity profiles^[144, 145]^. A common method for modeling the coronary microcirculation within each myocardial layer is to compartmentalize it into its principal functional constituents: arterioles, capillaries, and venules. Each compartment was represented by a lumped-parameter (0-D) model incorporating the three fundamental hemodynamic properties: viscous resistance (R), vascular compliance (C), and blood flow inertia (L). These individual 0-D models were then integrated to form a comprehensive network that simulates the hemodynamics of the entire vascular bed within each myocardial layer. This network model enforces the conservation of mass and momentum, with the governing equations solved at predefined pressure and flow nodes to ensure numerical stability and physiological fidelity^[146]^. Mynard et al. proposed a three-layer intracardiac vessel model coupled with a coronary artery main-branch vessel model, validating its reliability through animal experiments^[97]^. Other groups have also developed diverse coronary microcirculation models tailored to their specific research objectives^[147, 148]^.

At the pressure node, the conservation of mass equation is expressed as

|  | $\frac{dV}{dt}=Q_{j}-Q_{j+1}$ | (7) |
| --- | --- | --- |

At the flow node, the momentum conservation equation is expressed as

|  | $L_{j}\frac{dQ_{j}}{dt}=P_{j-1}-Q_{j}R_{j}-P_{j}$ | (8) |
| --- | --- | --- |

Herein, *P*_j_ and *P*_j-1_ respectively represent the pressure at the *C*_j_ and *C*_j-1_ compartment; *t* represents time; *Q*_j_ and *Q*_j+1_ respectively represent the inflow and outflow of traffic from the *C*_j_ compartment; *R*_j_ and *L*_j_ respectively represent the impedance and inertia coefficient connecting *C*_j_ and *C*_j-1_.

Table.A Summary of Abbreviations

| Full term | Abbreviation |  | Full term | Abbreviation |
| --- | --- | --- | --- | --- |
| Cardiovascular diseases | CVDs |  | fluid-solid interaction | FSI |
| Coronary artery disease | CAD |  | three-dimensional | 3-D |
| percutaneous coronary intervention | PCI |  | zero-dimensional | 0-D |
| coronary artery bypass grafting | CABG |  | one-dimensional | 1-D |
| coronary flow reserve | CFR |  | two-dimensional | 2-D |
| fractional flow reserve | FFR |  | quantitative flow ratio | QFR |
| positron emission tomography | PET |  | virtual flow reserve fraction | vFFR |
| artificial intelligence | AI |  | Virtual Functional Assessment Index | vFAI |
| computational fluid dynamics | CFD |  | optical coherence tomography-based fractional flow reserve | OFR |
| coronary computed tomography angiography | CCTA |  | ultrasonic flow ratio | UFR |
| digital subtraction angiography | DSA |  | machine learning | ML |
| optical coherence tomography | OCT |  | deep learning | DL |
| intravascular ultrasound | IVUS |  | physics-informed neural networks | PINN |
| Thrombolysis in Myocardial Infarction | TIMI |  | lumped parameter model | LPM |
| single photon emission computed tomography imaging | SPECT |  | stenosis flow resistance | SFR |
| computed tomography perfusion | CTP |  | invasive coronary angiography | ICA |
| and magnetic resonance | MR |  | long short-term memory | LSTM |
| instantaneous wave-free ratio | iFR |  | support vector machine | SVM |
| transcatheter aortic valve implantation | TAVI |  | degree of stenosis | DS |
| coronary microvascular resistance | CMVR |  | left ventricular myocardium | LVM |
| myocardial infarction | MI |  | Arbitrary Lagrangian–Eulerian | ALE |

References

[12] Taylor CA, Fonte TA, Min JK. Computational fluid dynamics applied to cardiac computed tomography for noninvasive quantification of fractional flow reserve: scientific basis. J Am Coll Cardiol. 2013;61(22):2233-41.

[86] Liu B, Zheng J, Bach R, Tang D. Influence of model boundary conditions on blood flow patterns in a patient specific stenotic right coronary artery. BioMed Eng OnLine. 2015;14(Suppl 1)

[97] Mynard JP, Penny DJ, Smolich JJ. Scalability and in vivo validation of a multiscale numerical model of the left coronary circulation. Am J Physiol Heart Circ Physiol. 2014;306(4):H517-H28.

[127] Kim HJ, Vignon-Clementel IE, Coogan JS, Figueroa CA, Jansen KE, Taylor CA. Patient-specific modeling of blood flow and pressure in human coronary arteries. Ann Biomed Eng. 2010;38(10):3195-209.

[128] Min JK, Leipsic J, Pencina MJ, Berman DS, Koo B-K, van Mieghem C, et al. Diagnostic accuracy of fractional flow reserve from anatomic CT angiography. JAMA. 2012;308(12):1237-45.

[129] Nørgaard Bjarne L, Leipsic J, Gaur S, Seneviratne S, Ko Brian S, Ito H, et al. Diagnostic performance of noninvasive fractional flow reserve derived from coronary computed tomography angiography in suspected coronary artery disease: the NXT trial (Analysis of Coronary Blood Flow Using CT Angiography: Next Steps). J Am Coll Cardiol. 2014;63(12):1145-55.

[130] Gashi K, Bosboom EMH, van de Vosse FN. The influence of model order reduction on the computed fractional flow reserve using parameterized coronary geometries. J Biomech. 2019;82313-23.

[131] Ghigo AR, Fullana J-M, Lagrée P-Y. A 2D nonlinear multiring model for blood flow in large elastic arteries. J Comput Phys. 2017;350136-65.

[132] Guan D, Liang F, Gremaud PA. Comparison of the Windkessel model and structured-tree model applied to prescribe outflow boundary conditions for a one-dimensional arterial tree model. J Biomech. 2016;49(9):1583-92.

[133] Liang FY, Takagi S, Himeno R, Liu H. Biomechanical characterization of ventricular-arterial coupling during aging: a multi-scale model study. J Biomech. 2009;42(6):692-704.

[134] Blanco PJ, Watanabe SM, Passos MA, Lemos PA, Feijoo RA. An anatomically detailed arterial network model for one-dimensional computational hemodynamics. IEEE Trans Biomed Eng. 2015;62(2):736-53.

[135] Blanco PJ, Watanabe SM, Dari EA, Passos MARF, Feijóo RA. Blood flow distribution in an anatomically detailed arterial network model: criteria and algorithms. Biomech Model Mechanobiol. 2014;13(6):1303-30.

[136] Mynard JP, Smolich JJ. Influence of anatomical dominance and hypertension on coronary conduit arterial and microcirculatory flow patterns: a multiscale modeling study. Am J Physiol Heart Circ Physiol. 2016;311(1):H11-H23.

[137] Ahmad S, Zeb S, Sun Y, Guo H, Qiao Y. Non-Newtonian rheological aspects of blood in computational hemodynamics. Phys Rep. 2026;11681-24.

[138] Gow BS, Hadfield CD. The elasticity of canine and human coronary arteries with reference to postmortem changes. Circ Res. 1979;45(5):588-94.

[139] Formaggia L, Nobile F, Quarteroni A, Veneziani A. Multiscale modelling of the circulatory system: a preliminary analysis. Comput Visual Sci. 1999;2(2):75-83.

[140] Shi Y, Lawford P, Hose R. Review of zero-D and 1-D models of blood flow in the cardiovascular system. Biomed Eng Online. 2011;10(1):33.

[141] Young DF, Tsai FY. Flow characteristics in models of arterial stenoses. I. Steady flow. J Biomech. 1973;6(4):395-410.

[142] Seeley BD, Young DF. Effect of geometry on pressure losses across models of arterial stenoses. J Biomech. 1976;9(7):439-48.

[143] Huo Y, Svendsen M, Choy JS, Zhang ZD, Kassab GS. A validated predictive model of coronary fractional flow reserve. J R Soc Interface. 2012;9(71):1325-38.

[144] Schrauwen JT, Wentzel JJ, van der Steen AF, Gijsen FJ. Geometry-based pressure drop prediction in mildly diseased human coronary arteries. J Biomech. 2014;47(8):1810-5.

[145] Schrauwen JTC, Koeze DJ, Wentzel JJ, van de Vosse FN, van der Steen AFW, Gijsen FJH. Fast and accurate pressure-drop prediction in straightened atherosclerotic coronary arteries. Ann Biomed Eng. 2015;43(1):59-67.

[146] Alastruey J, Parker KH, Peiro J, Sherwin SJ. Lumped Parameter Outflow Models for 1-D Blood Flow Simulations:Effect on Pulse Waves and Parameter Estimation. Commun Comput Phys. 2008;4(2):317-36.

[147] van der Horst A, Boogaard FL, van′t Veer M, Rutten MCM, Pijls NHJ, van de Vosse FN. Towards patient-specific modeling of coronary hemodynamics in healthy and diseased state. Comput Math Methods Med. 2013;2013393792.

[148] Nicolas PS. A computational study of the interaction between coronary blood flow and myocardial mechanics. Physiol Meas. 2004;25(4):863-77.
